# The impact of maternal antenatal treatment with two doses of azithromycin and monthly sulphadoxine-pyrimethamine on child weight, mid-upper arm circumference and head circumference: A randomized controlled trial

**DOI:** 10.1371/journal.pone.0216536

**Published:** 2019-05-07

**Authors:** Lotta Hallamaa, Yin Bun Cheung, Mari Luntamo, Ulla Ashorn, Teija Kulmala, Charles Mangani, Per Ashorn

**Affiliations:** 1 Center for Child Health Research, Faculty of Medicine and Health Technology, Tampere University, Tampere, Finland; 2 Center for Quantitative Medicine, Duke-NUS Medical School, Singapore; 3 Department of Public Health, School of Public Health and Family Medicine, College of Medicine Malawi, Blantyre, Malawi; 4 Department of Paediatrics, Tampere University Hospital, Tampere, Finland; Centers for Disease Control and Prevention, UNITED STATES

## Abstract

**Aim:**

Intermittent preventive treatment in pregnancy (IPTp) with azithromycin and monthly sulfadoxine-pyrimethamine increased the mean child weight, mid-upper arm and head circumference at four weeks of age in a rural low-income setting. Now we assess for how long these gains were sustained during 0–5 years of age.

**Methods:**

We enrolled 1320 pregnant Malawian women in a randomized trial and treated them with two doses of sulfadoxine-pyrimethamine (control) or monthly sulfadoxine-pyrimethamine as IPTp against malaria, or monthly sulfadoxine-pyrimethamine and two doses of azithromycin (AZI-SP) as IPTp against malaria and reproductive tract infections. Child weight, mid-upper arm circumference, head circumference and weight-for-height Z-score were recorded at one, six, 12, 24, 36, 48, and 60 months.

**Results:**

Throughout follow-up, the mean child weight was approximately 100 g higher (difference in means 0.12 kg, 95% CI 0.04–0.20, P = 0.003 at one month; 0.19 kg, 95% CI 0.05–0.33, P = 0.007, at six months), mean head circumference 2 mm larger (0.3 cm, 95% CI 0.1 to 0.5, P = 0.004 at one month) and the cumulative incidence of underweight by five years of age was lower (hazard ratio 0.74, 95% CI 0.60 to 0.90, P = 0.002) in the AZI-SP group than in the control group. The 2 mm difference in the mean mid-upper arm circumference at one month (0.2 cm, 95% CI 0.0 to 0.3, P = 0.007) disappeared after three years of age. There was no difference in mean weight-for-height Z-score at any time point.

**Conclusion:**

In Malawi, IPTp with azithromycin and monthly sulfadoxine-pyrimethamine has a modest, 3-5-year positive impact on child weight, mid-upper arm circumference and head circumference, but not on weight-for-height Z-score.

## Introduction

Childhood growth failure is common, especially in sub-Saharan Africa and in southern Asia. We have previously reported results from the “Lungwena Antenatal Intervention Study” (LAIS), that suggest that birth and neonatal size can be increased with intensive maternal infection control during pregnancy[1,2]. We have also shown that the length gain attained during fetal period is sustained during the first five years of life[3]. Results of the follow-up study thus suggested that increased length in bone is not lost during childhood. However, the situation might be different with child weight, mid-upper arm circumference (MUAC) and head circumference (HC). This is suggested by results from studies using maternal dietary supplementation with energy and/or nutrients, where fetal-period gains have largely been lost within a year or two after birth[4,5].

The aim of the current study was to determine for how long the gains in child weight, MUAC and HC obtained during pregnancy are sustained during the five-year follow-up among children born to mothers treated with monthly SP and two doses of azithromycin as compared to children born to women in the control group.

## Methods

### Study design

This study was a five-year follow-up to the LAIS, a single-center, randomized, partially placebo controlled, outcome assessor-blinded, three-arm clinical trial conducted in rural Malawi[1]. The main outcomes of the original trial were the incidence of preterm delivery, low birth weight and infant size at one month[1,2]. Primary outcomes of the follow-up study were child length/height and stunting throughout the five-year follow-up, total developmental score at five years, and the total number of deaths. These results have been published earlier and they supported a hypothesis that the provision of AZI-SP rather than two doses of SP during pregnancy reduced the incidence and prevalence of childhood stunting, had a positive effect on child development, and may have reduced postneonatal mortality[3]. This paper reports the trends in child weight, MUAC and HC in the three study groups during the five-year follow-up.

### Participants and follow-up

Details of the inclusion and exclusion criteria, and randomization are available in the original trial publication[1]. In brief, we randomly allocated 1320 women to either a control group or to one of two intervention groups: monthly SP or AZI-SP. Women in the control group received standard Malawian antenatal care, which at the time of the study included intermittent preventive treatment in pregnancy with SP (three tablets orally, each containing 500 mg of sulfadoxine and 25 mg of pyrimethamine) twice: at enrollment and between 28–34 weeks of gestation. At these visits, they also received a placebo in lieu of azithromycin. Women in the monthly SP group received SP monthly from enrollment until 37 gestational weeks and two doses of placebo in lieu of azithromycin. Women in the AZI-SP group received monthly SP and active azithromycin (two tablets orally, each containing 500 mg of azithromycin) twice: at enrollment and between 28–34 weeks of gestation. Both human immunodeficiency virus (HIV) negative and positive mothers were enrolled to the study. Only participants who signed or thumb-printed an informed consent form were enrolled in the study.

All children received standard Malawian care during follow-up; HIV positive mothers and their newborns received nevirapine during/immediately after delivery for prevention of mother-to-child transmission of HIV.

Child anthropometrics were assessed at the study clinic visits at one, six, 12, 24, 36, 48 and 60 months after birth. Anthropometrists measured child weight with a SECA scale (SECA 834, Chasmors Ltd; reading increment of 10 g), but between 36 and 60 months the weight of some children was occasionally measured erroneously with a bathroom scale (reading increment of 1 kg; please see analytic strategy below). Anthropometrists measured child length (≤24 months) with a length board (Kiddimetre, Raven Equipment Ltd, reading increment of 1 mm) or height (>24 months) with a Harpenden stadiometre (Holtain Limited, reading increment of 1 mm). Arm and head circumferences were measured with a non-elastic plastic tape (Lasso-o tape, Harlow Printing Limited; reading increment of 1 mm). Research personnel assessing child anthropometrics were different from those who gave the mother the pre-packed study drugs. Anthropometrists were not involved in data analysis and they remained blinded to the intervention group allocation throughout the follow-up.

Both the original trial and the follow-up were performed according to Good Clinical Practice and ethical standards of Declaration of Helsinki. The protocol was approved by the College of Medicine Research and Ethics Committee, Malawi (original trial on Apr 30, 2003 and the follow-up on Feb 22, 2006) and the Ethical Committee of Pirkanmaa Hospital District, Finland (original trial on Apr 29, 2003 and the follow up on May 9, 2006). The trial was registered with the U.S. National Library of Medicine (http://www.clinicaltrials.gov) with trial identification NCT00131235 on Aug 17, 2005. Because the trial started enrollment before Jul 1, 2005, when the registration of clinical trials became a requirement, the trial was not registered before the start of enrollment[6]. The authors confirm that all ongoing and related trials for this drug/intervention are registered.

### Outcomes

In this paper we report the secondary growth outcomes of the follow-up study that were child weight, MUAC, and HC at one, six, 12, 24, 36, 48 and 60 months of age. Child size at one month of age has been reported earlier[2] but we included those results in this manuscript for consistency and ease of reading the results. Small differences in the results at one month of age in this manuscript compared to those reported earlier[2] are due to different time frame in which the measurement was included in the analysis. If the child did not come for a scheduled study clinic visit, the study team traced and interviewed the caretaker, and completed a structured verbal autopsy questionnaire in case the child had died. Number of child deaths during the follow-up have been reported earlier[3]. None of the deaths were judged to be due to the maternal intervention.

For anthropometric measurements before 24 months of age, we considered data missing if the actual date of measurement was off by more than one month from the target date. For anthropometric measurements at or after 24 months of age, we considered data missing if the actual date of measurement was off by more than two months from the target date.

We calculated age- and sex-standardized weight-for-age Z-score (WAZ), weight-for-height Z-score (WHZ), MUAC-for-age Z-score (MUACZ) and HC-for-age Z-score (HCZ) using the World Health Organization Child Growth Standards[7,8]. Growth standards allow comparison of children to a healthy growing population regardless of ethnicity, socio-economic status and other background information[7]. As per the Standards, MUACZ is available only from three months of age onwards[7] and there is no WHZ, MUACZ, or HCZ WHO reference for children of 60 months of age and older[8]. Thus only WAZ could be analyzed for children measured between 60–62 months and WAZ, WHZ, MUACZ, and HCZ were analyzed for children measured between 58–60 months. Values below -2 Z-scores were considered underweight, wasting, low MUAC, and small HC. Anthropometrics at their original metrics were analyzed even though reference was not available for conversion to Z-score.

The associations between intervention and outcome variables are primarily shown without covariate adjustment, as per predefined statistical analysis plan and International guidelines[9,10] that highlight the importance of adhering to the statistical analysis plan when presenting main results.

### Statistical analysis

The sample size of 440 pregnant women per group was planned to give 80% power at a 5% level of significance to detect a 40% reduction in the rate of preterm delivery which was the trial’s main hypothesis[1].

Group codes for the study were broken for the analysis of trial’s main hypothesis. Statistician for this follow-up was different than the one doing the analyses for the main hypothesis. For this follow-up study the statistician (LH) merged the intervention code with follow-up data only after data was cleaned, analysis plan written and the syntax for the analysis done with a mock code. The analysis was based on the principle of intention-to-treat. We conducted statistical analyses with Stata 13.1 (StataCorp, College Station, USA).

For absolute weight, MUAC, HC, and Z-scores we calculated group means and used least squares regression to estimate differences between groups. For prevalence of underweight, wasting, low MUAC, and small HC we calculated percentages and used log-binomial regression model to estimate risk ratios or used a modified Poisson regression in case the log-binomial regression did not converge[11]. We used competing-risks regression[12] to estimate cumulative incidence of underweight, wasting, low MUAC, and small HC under the competing risk of death.

We took intragroup correlation due to twin pregnancies into account by using robust standard errors for clustered data[13]. To prevent inflated type I errors due to testing between multiple groups, we began hypothesis testing with a global null hypothesis of no difference between any groups (Closed Testing Procedure)[14]. Pairwise null hypotheses were rejected only if the global null hypothesis was also rejected. We rejected a null hypothesis if two-sided P<0.05.

A number of sensitivity analyses were performed: an analysis with multiple imputation for missing data for growth outcomes[15]; an analysis with adjustment for maternal malaria at enrollment, HIV status, height, body mass index, number of previous pregnancies, number of school years, and child sex, selected based on predefined criteria presented in the statistical analysis plan; and an analysis with multiply imputed data to replace weight measurements rounded to the full kilogram. Because no record was made of weight measurements that were taken with bathroom scales, it was not possible to identify participants with weight rounded to the nearest kilogram. As a sensitivity analysis, we considered all full kilogram weight measurements recorded at or after 36 months as censored within an interval of ± 0.5 kg of the full kilogram and used multiple imputation with interval censoring to replace the value. We performed a post-hoc analysis of anthropometric measurements at last time point for which they were available by maternal malaria and HIV status. Because of differences in time of last available measurement, we used Z-scores in this analysis.

## Results

Between December 1, 2003 and October 11, 2006 1320 women were enrolled to the study and randomized to control (436), monthly SP (441) and AZI-SP groups (443). At enrollment the intervention groups were similar except for small differences in the prevalence of malaria parasitemia and mean number of previous pregnancies (S1 Table).

**Fig 1:**
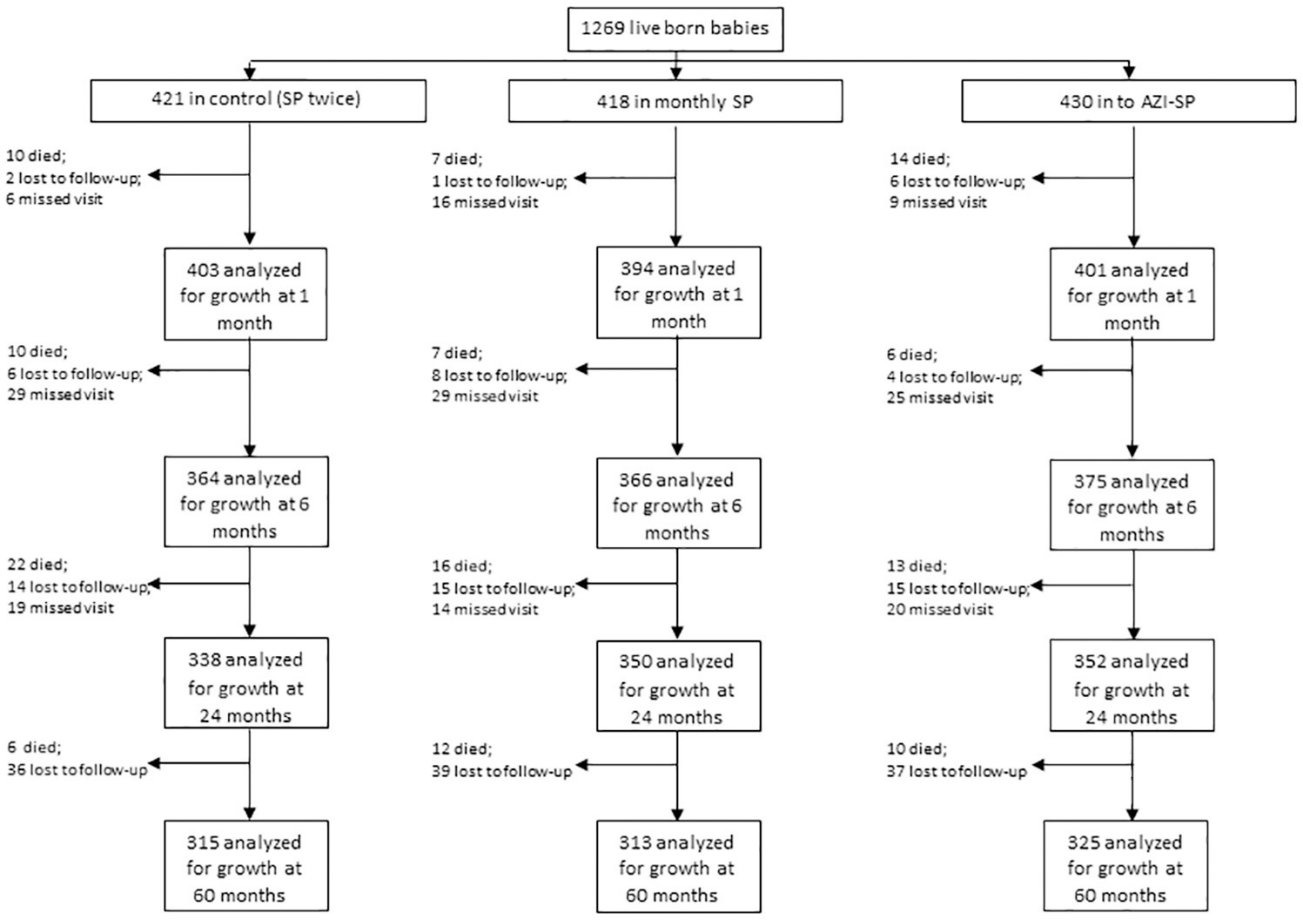
Follow-up of liveborn children. SP = sulfadoxine-pyrimethamine; AZI-SP = intervention group with monthly SP and two doses of azithromycin.

Four mothers were lost to follow-up during pregnancy and 1269 of 1327 (95.6%) babies were born alive. The last follow-up visit was completed on March 16, 2012. Data was available from 1040 (82.0%) participants at 24 months and 953 (75.1%) participants at 60 months of age (Fig 1). Success of follow-up was similar between the intervention groups (Fisher’s exact test P = 0.44 and P = 0.97 for proportions with anthropometric measurements at 24 and 60 months, respectively). At enrollment the intervention groups had similar maternal background characteristics except for small differences in the prevalence of malaria parasitemia and proportion of primiparous women (Table 1 and S1 Table). At birth, mean duration of pregnancy was longer in the AZI-SP group compared to the control group. Children born to mothers in the AZI-SP group had higher birth weight compared to those in the control and monthly SP groups (Table 1).

**Table 1:** Baseline characteristics of the participating women at enrollment and infant characteristics at birth for liveborn children, by study group.

Characteristic	Control (SP twice) (N = 421), n (%)	Monthly SP, (N = 418), n (%)	AZI-SP (N = 430), n (%)
**Maternal characteristics**			
Age, years, mean (SD)	25 (7)	25 (7)	25 (6)
Gestational age at enrollment, weeks, mean (SD)	20.3 (3.0)	20.0 (3.2)	20.0 (3.0)
Primiparous	104 (24.7%)	101 (24.2%)	88 (20.5%)
HIV positive	46/384 (12.0%)	62/376 (16.5%)	46/385 (12.0%)
Microsopic peripheral blood malaria parasitemia	46/420 (11.0%)	38/418 (9.1%)	25/430 (5.8%)
**Infant characteristics**			
Duration of pregnancy, mean (SD)	38.5 (2.0) (1 missing data)	38.7 (2.0)	38.9 (1.8) (3 missing data)
Birth weight, kg, mean (SD)	2.89 (0.47) (18 missing data)	2.96 (0.48) (27 missing data)	3.02 (0.45) (24 missing data)
Low birth weight, <2.5 kg	52/402 (12.9%)	36/391 (9.2%)	32/406 (7.9%)
Small for gestational age^a^	84/402 (20.9%)	72/389 (18.5%)	67/404 (16.6%)

SP = sulfadoxine-pyrimethamine. AZI-SP = intervention group with monthly SP and two doses of azithromycin. BMI = body-mass index. HIV = human immunodeficiency virus. Hb = hemoglobin.

^a^ Small for gestational age calculated using INTERGROWTH-21^st^ Project standards for newborn size by gestational age, defined as birthweight-for-gestation-week < 10^th^ centile.

Maternal background characteristics were mostly similar between children lost to follow-up by 60 months and those who remained in the study (S2 Table). Duration of pregnancy at enrollment and at birth was 0.4 weeks shorter among children who remained in the study than those who were lost to follow up by 60 months of age (P = 0.04 and P<0.01 for duration of pregnancy at enrollment and at birth, respectively). The proportion of HIV-positive mothers was lower among children who remained in the study than among those who were lost to follow-up (P<0.01). Birth weight was lower (P<0.01) and proportion of low birth weight higher (P = 0.02) among children who were lost to follow-up compared to those who remained in the study.

The median number of scheduled SP treatments received by the mothers of liveborn babies was 2 in the control group, 4 in the monthly SP group, and 4 in the AZI-SP group. Women in the AZI-SP group received a median of 2 azithromycin doses.

**Fig 2:**
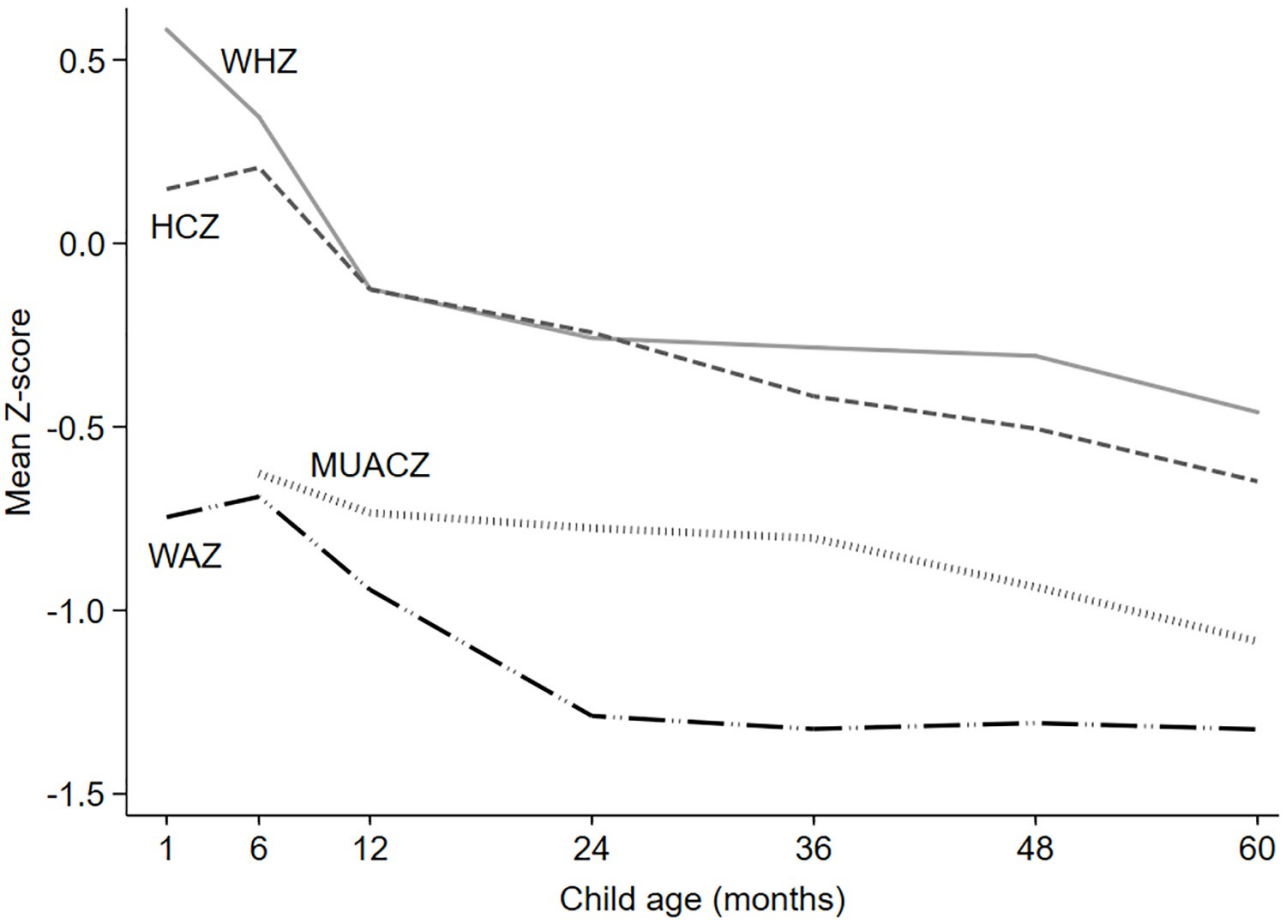
Mean weight-for-age Z-score, weight-for-height Z-score, mid-upper arm-circumference-for-age Z-score, and head circumference-for-age Z-score. At one, six, 12, 24, 36, 48 and 60 months of age, all intervention groups combined. WAZ = weight-for-age Z-score, WHZ = weight-for-height Z-score, MUACZ = mid-upper arm-circumference-for-age Z-score, HCZ = head circumference-for-age Z-score.

The mean absolute weight of children in the study cohort was 7.1 kg at 6 months and 15.4 kg at 60 months, corresponding to a mean WAZ of -0.69 and -1.32, respectively (Fig 2). The mean WHZ was 0.34 at 6 months and -0.46 at 60 months. The mean MUAC at the respective time points was 13.4 cm and 15.2 cm, and MUACZ -0.63 and -1.08. The mean HC was 43.1 cm at 6 months and 49.4 cm at 60 months, corresponding to a mean HCZ of 0.21 and -0.65, respectively.

**Fig 3:**
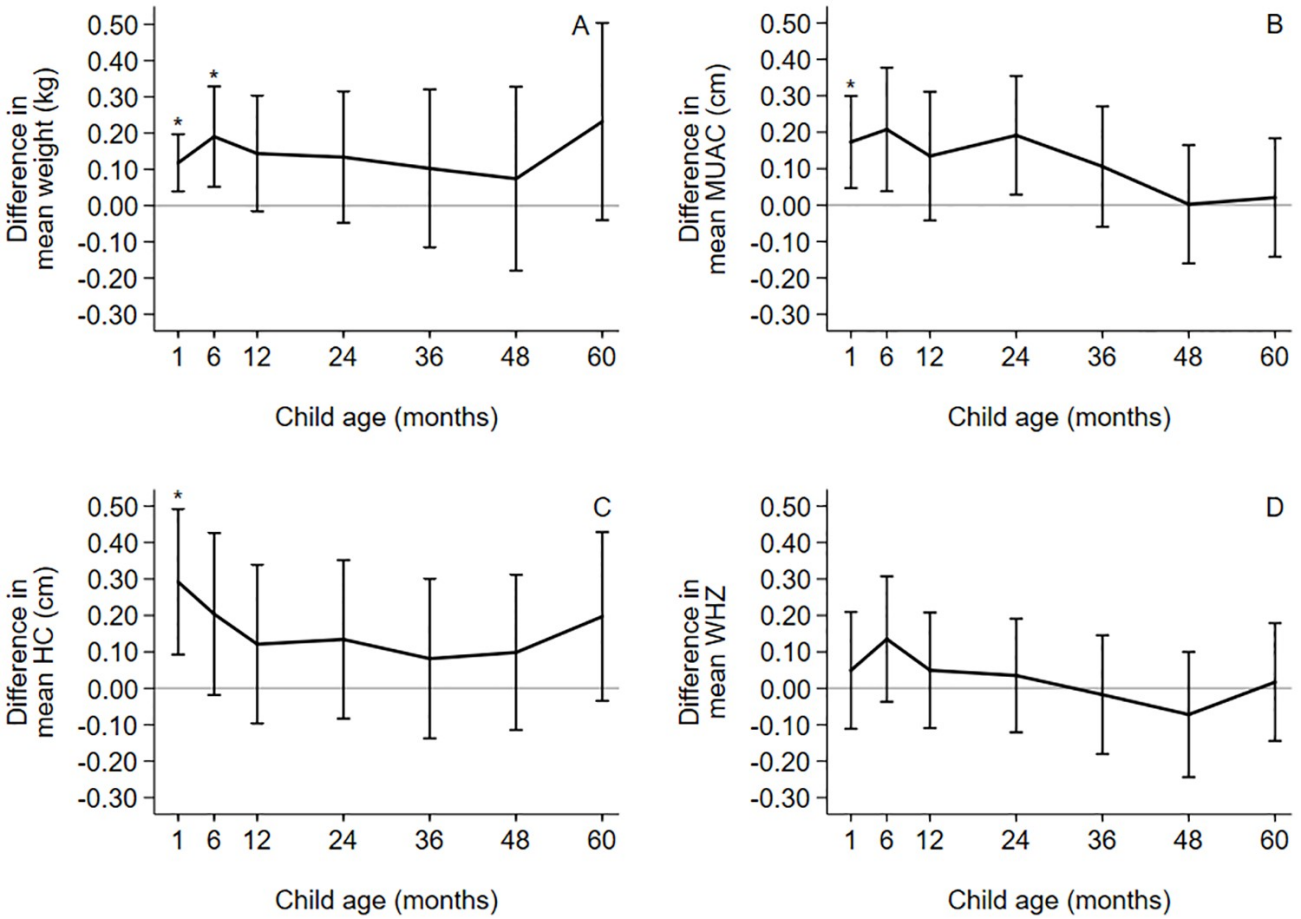
Differences between groups and 95% confidence interval for AZI-SP vs control in growth outcomes. (A) Mean weight (kg). (B) Mean mid-upper arm circumference (cm). (C) Mean head circumference (cm). (D) Mean weight-for-height Z-score (WHZ). SP = sulfadoxine-pyrimethamine; AZI-SP = intervention group with monthly SP and two doses of azithromycin. * denotes global P<0.05.

Mean absolute weight was 74–232 g higher in the AZI-SP group than in the control group at different time-points during the follow-up. The differences were statistically significant at one (0.12 kg, 95% CI 0.04–0.20, P = 0.003) and at six months (0.19 kg, 95% CI 0.05–0.33, P = 0.007) (Fig 3A, S3 Table). After covariate adjustment the mean difference in weight between the AZI-SP and the control group varied between 81–224 g with statistically significant differences at one (0.08 kg, 95% CI -0.01 to 0.16, P = 0.034) and at six months (0.16 kg, 95% CI 0.02 to 0.29, P = 0.023) (S3 Table). Unadjusted and covariate adjusted mean absolute MUAC was 1–2 mm higher in the AZI-SP group as compared to the control group until 36 months and was equal to the control group from 48 to 60 months (unadjusted difference between groups at one month 0.2 cm, 95% CI 0.0 to 0.3, P = 0.007) (Fig 3B, S4 Table). Mean absolute HC was 1–3 mm larger in the AZI-SP group than in the control group throughout the follow-up (difference between groups at one month 0.3 cm, 95% CI 0.1 to 0.5, P = 0.004) (Fig 3C, S5 Table). After covariate adjustment the mean difference varied between 0–2 mm with statistically significant differences at one (0.2 cm, 95% CI 0.0 to 0.4, P = 0.018) and at six months (0.2 cm, 95% CI 0.0 to 0.4, P = 0.048) (S5 Table). Differences between the groups in mean WHZ were not consistently in favor of AZI-SP or any other intervention group over time (Fig 3D, S3 Table). The results were similar after covariate adjustment (S3 Table). Differences in means between the monthly SP and the control group had the same pattern as the AZI-SP versus control group but the differences were smaller and statistically not significant (S1 Fig, S3–S5 Tables). In general, group means of monthly SP were between the mean values of the AZI-SP and the control groups, but differences between monthly SP and either of the other groups were not statistically significant (S1 Fig, S3–S5 Tables).

**Fig 4:**
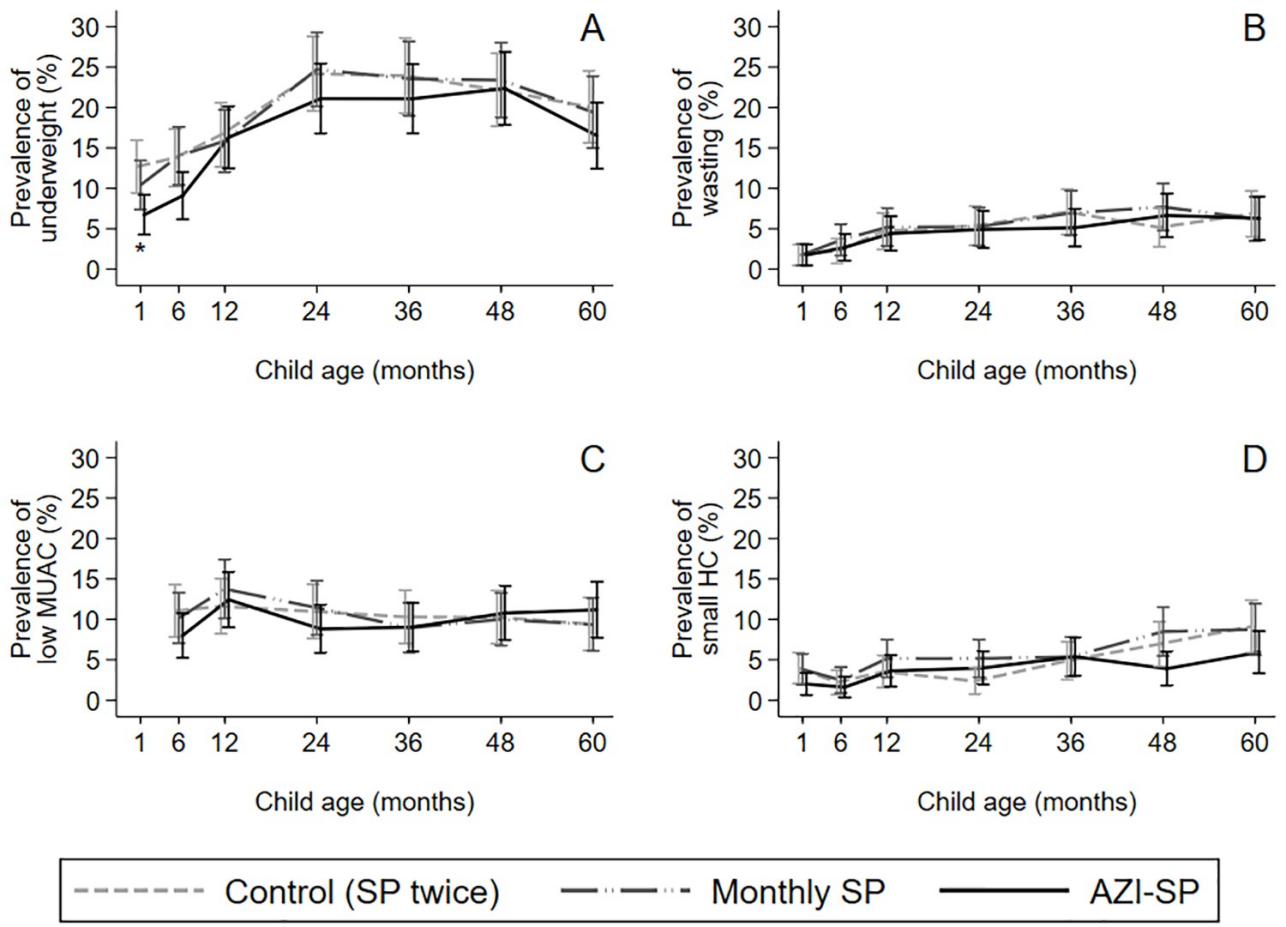
Prevalence of underweight, wasting, low mid-upper arm circumference, and small head circumference by intervention group. At one, six, 12, 24, 36, 48 and 60 months of age. (A) Underweight defined as weight-for-age Z-score < -2. (B) Wasting defined as weight-for-height Z-score (WHZ) < -2. (C) Low mid-upper arm circumference (MUAC) defined as MUAC-for-age Z-score < -2. (D) Small head circumference (HC) defined as HC-for-age Z-score < -2. SP = sulfadoxine-pyrimethamine; AZI-SP = intervention group with monthly SP and two doses of azithromycin. * denotes P<0.05 between AZI-SP and the control group.

**Fig 5:**
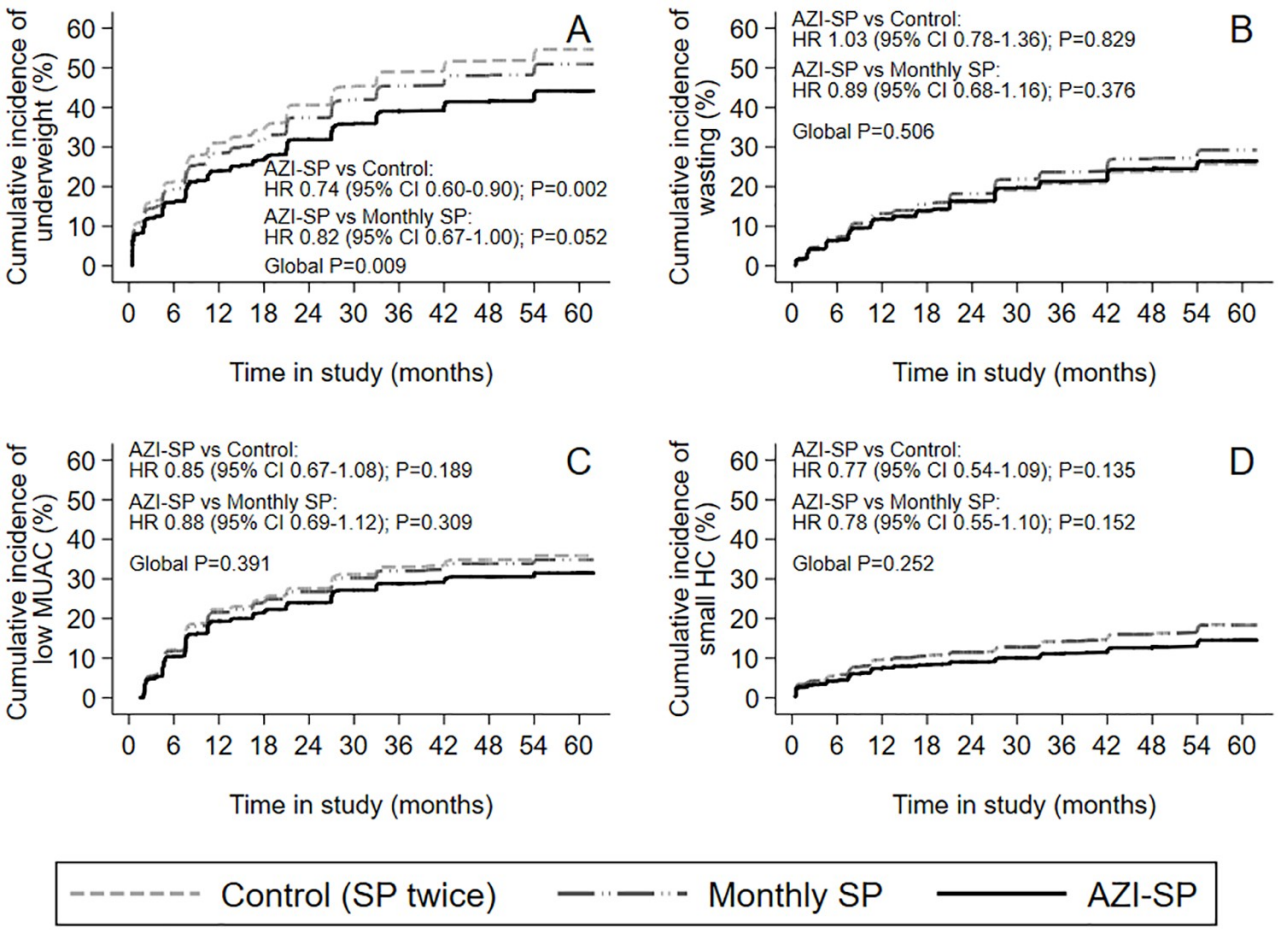
Cumulative incidence of underweight, wasting, low mid-upper arm circumference, and small head circumference. Under competing risk of death by 60 months of age by intervention group. (A) Underweight defined as weight-for-age Z < -2. (B) Wasting defined as weight-for-height Z (WHZ) < -2. (C) Low mid-upper arm circumference (MUAC) defined as MUAC-for-age Z < -2. (D) Small head circumference (HC) defined as HC-for-age Z < -2. SP = sulfadoxine-pyrimethamine; AZI-SP = intervention group with monthly SP and two doses of azithromycin; HR = hazard ratio. * denotes P<0.05 between AZI-SP and the control group.

There were no big differences between the groups in the prevalence of underweight, wasting, low MUAC, and small HC (Fig 4, S6 Table). There were no major differences between unadjusted and covariate adjusted results (S6 Table). Cumulative incidence of underweight by five years of age was statistically significantly lower in the AZI-SP group compared to the control group but there were no differences between the groups in the incidence of wasting, low MUAC and small HC (Fig 5).

Results from the sensitivity analyses with multiple imputed data to control for missing anthropometric data and to replace weight measurements rounded to the full kilogram, and covariate adjusted differences in means and risk ratios were consistent with those from the primary analyses (S3–S6 Tables). There were no differences in mean child WAZ, WHZ, MUACZ and HCZ between children whose mothers were HIV negative and HIV positive at enrollment (S7 Table). Among children whose mothers were malaria positive at enrollment, HCZ was 0.29 (P = 0.004) Z-scores lower compared to those whose mothers were malaria negative at enrollment. There were no differences in WAZ, WHZ and MUACZ (S7 Table).

## Discussion

In this study, we sought to analyze postnatal sustainability of fetal gains in weight, MUAC and HC, obtained through an intensified antenatal maternal infection management intervention. In our sample, children, whose mothers had received monthly SP and two doses of azithromycin during pregnancy rather than two SP doses had approximately 100 g higher mean weight and 2 mm larger mean head circumference throughout the five-year follow-up than the children in the control group. However, the difference was statistically significant only until six months with weight and until one month with head circumference. The mean MUAC was approximately 2 mm higher in the AZI-SP group than in the control group from one month until three years of age, with statistically significant difference at one month, but the difference disappeared after three years of age. There was no difference in the mean WHZ at any point of measurement. The cumulative incidence of underweight by five years of age was lower in the AZI-SP group than in the control group, whereas in the same timeframe there was no difference between the groups in the point-prevalence or cumulative incidence in wasting, low MUAC or small head circumference. Mean values of weight, MUAC and HC in monthly SP group were generally between AZI-SP and the control group but the differences were not statistically significant with either of the groups.

The validity of the sample findings is supported by random group allocation, broad inclusion criteria, large sample size, comprehensive follow-up, and blinding of the outcome assessors. Potential bias could have been introduced by missing data on anthropometric measurements and some weight measurements rounded to the nearest full kilogram at 36 months and thereafter. However, we implemented an active tracing system for defaulters and the random allocation was likely to distribute the non-trial determinants of growth evenly between the study groups. We have no record of participants who were weighed with bathroom scale at 1 kg increment but because the anthropometrists doing the measurements were blinded to the group allocation of the mothers, we believe this occasional use of bathroom scale was randomly allocated between intervention groups. Also, random measurement error in the outcome variable does not affect the estimate but is reflected in the standard error of the estimate, and can thus lead to under-estimation but not over-estimation of the statistical significance in difference between groups[16]. Furthermore, the baseline characteristics of those lost to follow-up and those who remained in the study were similar. Hence we believe that these factors did not bias our conclusions.

Due to the increasing population variance with age[17], intergroup differences in absolute size that are statistically significant at birth may not be so in older age groups. In the current sample, difference in weight was no longer statistically significant after six months and difference in MUAC and head circumference after one month. However, the point-estimates remained essentially constant for the absolute weight difference from birth to five years, for MUAC from birth to three years and fell only slightly for head circumference from birth to five years. Because of the constant inter-group difference, robustness of the results for sensitivity analyses, and the biological plausibility of the findings, we consider it likely that the population differences in weight and in head circumference are sustained beyond 1–6 months even though the sample differences were not statistically significant at later time points. The small fetal gains in MUAC in the AZI-SP group were sustained until three years of age but lost thereafter. No differences between the groups in weight-for-height were observed at any measurement point.

Although monthly SP has been shown to reduce low birth weight by 20% compared to two doses of SP during pregnancy[18], our results do not support the hypothesis that monthly SP would have a positive, sustained effect on child growth during the first five years of live. The mean values of weight, MUAC and HC in the monthly SP group tended to be higher than those in the control group but remained lower compared to the AZI-SP group, suggesting that monthly SP alone is not enough to create long-lasting effect on child growth.

We were not able to identify any other studies that would have tested the effect of antibiotic treatment during pregnancy on child growth after the neonatal period. However, other studies have tested the hypothesis that a nutritional intervention provided to the mother during pregnancy would have positive effects on child size at birth and child growth between one to eight years after birth[4,5,19–22]. Results of these studies are somewhat contradictory. Two studies conducted in Burkina Faso[5,20] one study done in Malawi[4] and one in Nepal[21] found higher weight and/or length at birth or soon after, among children born to mothers who received dietary supplementation with energy and/or nutrients. However, these differences were largely lost within 1–8 years after birth. In iron + folic acid + zinc group in the Nepalese study[21] and in a study done in East Java[22], differences in child size between the groups were seen only during or at the end of the follow-up, but not at birth. Only one study done in Nepal had differences between study groups in child weight both at birth and at the end of the follow-up at 2–3 years of age[19]. Despite the different prenatal interventions used in the studies, the differences in absolute size between groups in those studies which reported these outcomes were quite similar after 1–8 years of follow-up as observed in our study[3,19,21,22]

We have earlier shown that the differences in length between the AZI-SP and the control group are sustained during the five-year follow-up[3]. Given these earlier and the current results, it seems possible that fetal weight gain is retained if it is related to linear growth and bone accrual whereas it is harder to sustain if it represents ponderal growth and fat or other soft tissue deposition. This is plausible since fat and muscle mass (and hence MUAC and WHZ) reflect the child’s current nutritional status, whereas attained length (and hence LAZ and WAZ) is also a function of the child’s earlier growth and environment[23,24].

The current results and those obtained earlier from the same cohort[2,3] suggest that intensified maternal infection control during pregnancy can improve fetal linear growth, resulting in a sustained effect on infant and child length and possibly weight and HC, but not on weight-for-height or MUAC. It should be noted, however, that although WHZ and MUAC are indicators of the child’s current nutritional status, they are far from covering it comprehensively[23]. Furthermore, anthropometric assessment does not in any way measure child development or mental and social wellbeing, which are desirable outcomes that have earlier been associated with nutritional or infection control interventions in pregnancy[3,25–29]. Hence, although increases in weight, MUAC and HC did not show statistically significant differences beyond six months, these results should not be interpreted as a discouraging indication that antenatal infection control or nutritional interventions could not produce long-standing “healthy growth” in the offspring[30]. It is quite possible that they can–but their assessment will require outcome measurement that goes beyond traditional anthropometrics.

## Supporting information

S1 Checklist CONSORT checklist. (DOC)

S1 Protocol Trial protocol. (PDF)

S1 Table Baseline characteristics of all women enrolled to the study at enrollment, by study group. (DOCX)

S2 Table Baseline characteristics of participating women at enrolment and infant characteristics at birth, by follow-up status at 60 months. (DOCX)

S3 Table Mean (SD) weight, weight-for-age Z-score (WAZ), and weight-for-height Z-score (WHZ) by intervention group at one, six, 12, 24, 36, 48, and 60 months of age. (DOCX)

S4 Table Mean (SD) mid-upper arm-circumference (MUAC) and mid-upper arm circumference-for-age Z-score (MUACZ) by intervention group at one, six, 12, 24, 36, 48, and 60 months of age. (DOCX)

S5 Table Mean (SD) head circumference (HC) and head circumference-for-age Z-score (HCZ) by intervention group at one, six, 12, 24, 36, 48, and 60 months of age. (DOCX)

S6 Table Prevalence of underweight, wasting, low mid-upper arm-circumference, and small head circumference by intervention group at one, six, 12, 24, 36, 48, and 60 months of age. Underweight defined as weight-for-age Z-score (WAZ) < -2. Wasting defined as weight-for-height Z-score (WHZ) < -2. Low mid-upper arm circumference (MUAC) defined MUAC-for-age Z-score (MUACZ) < -2. Small head circumference (HC) defined as HC-for-age Z-score < -2.
(DOCX)

S7 Table Mean (SD) weight-for-age Z-score (WAZ), weight-for-height Z-score (WHZ), mid-upper arm-circumference-for-age Z-score (MUACZ) and head circumference-for-age Z-score (HCZ) at last available time point by maternal malaria and HIV status at enrollment. (DOCX)

S1 Fig Differences between groups and 95% confidence interval for monthly SP and control groups and AZI-SP and monthly SP groups in mean weigh, mid-upper arm circumference, head circumference, and weight-for-height Z-score. (A) Mean weight (kg). (B) Mean mid-upper arm circumference (MUAC). (C) Mean head circumference (HC). (D) Mean weight-for-height Z-score (WHZ). SP = sulfadoxine-pyrimethamine; AZI-SP = intervention group with monthly SP and two doses of azithromycin. * denotes global P<0.05.
(TIF)
