# Supplementary material for: The impact of maternal antenatal treatment with two doses of azithromycin and monthly sulphadoxine-pyrimethamine on child weight, mid-upper arm circumference and head circumference: A randomized controlled trial
Source: PLoS One. 2019 May 7;14(5):e0216536. doi: 10.1371/journal.pone.0216536 (PMC6504037; doi:10.1371/journal.pone.0216536)
Supplement: S1 Protocol — (PDF) [file pone.0216536.s002.pdf]

Lungwena Antenatal Intervention Study, LAIS

A research plan for a single-centre intervention trial in rural Malawi, testing the maternal and infant health effects of presumptive intermittent treatment of pregnant women with sulfadoxine-pyrimethamine and azithromycin

Investigators (in alphabetical order)

Dr. Per Ashorn, MD[1, 2]

Dr. Chiwoza Bandawe, PhD[1]

Dr. Teija Kulmala, MD[2]

Dr. Mari Luntamo, MD[2]

Dr. Kenneth Maleta, MBBS[1]

Dr. Bernard Mbewe, MD[1]

Participating Academic Institutions

[1]Department of Community Health, College of Medicine, University of Malawi

&

[2]Paediatric Research Centre, Tampere University Medical School, Finland

Contact information: Dr. Per Ashorn, MD, University of Tampere Medical School, Paediatric Research Centre, FIN-33014 University of Tampere, Finland, Tel.: +358 40 7280 354, FAX: +358 3 215 8420, Email: per.ashorn@uta.fi

Table of contents

1. SUMMARY OF THE STUDY 3
1.1 FUNDING, MONITORING, AND RESEARCH SITES 3
1.2 EXECUTIVE SUMMARY 4
2. BACKGROUND 5
2.1 INTRODUCTION 5
2.2 JUSTIFICATION FOR MAKING THE STUDY NOW 5
2.3 OBJECTIVES OF THE STUDY 7
3. METHODS FOR THE STUDY 8
3.1 GENERAL DESIGN 8
3.2 PLACE OF RESEARCH 8
3.3 STUDY POPULATION 8
3.4 STUDY PERIOD 10
3.5 SAMPLE SIZE, ITS JUSTIFICATION, AND RECRUITEMENT RATE 11
3.6 DATA COLLECTION 12
3.7 DATA MANAGEMENT AND ANALYSIS 18
4. PRESENTATION, DISSEMINATION AND USE OF THE RESULTS 20
5. ETHICAL CONSIDERATIONS 20
5.1 INFORMED CONSENT 21
5.2 POSSIBLE RISKS TO THE SAFETY OF PARTICIPANTS INVOLVED IN THE STUDY 21
5.3 REVIEW BOARD 22
5.4 STUDY DISCONTINUATION CRITERIA 22
5.5 POTENTIAL PUBLIC HEALTH CONSEQUENSES OF THE STUDY 23
6. POSSIBLE CONSTRAINTS 24
7. TRAINING PROVIDED 24
8. BUDGET AND ITS JUSTIFICATION 25
9. REFERENCES 26
10. APPENDIXES 30

1. Summary of the study

1.1 Funding, monitoring, and research sites

Sponsor:	Investigator initiated study, funded by a research grant from the Academy of Finland (grant 79 787)
Monitor:	No external monitor besides the review board
Review board:	An independent body, overseeing the progress of the study and assessing the safety of the intervention<br><br>Dr. Michael Boele van Hensbroek, MD<br>Wellcome Trust Research Laboratories, Blantyre, Malawi<br>Tel.: +265 676444, FAX: +254 675774<br>Mobile: +265 9 926906, Email: mbvh@mlw.medcol.mw<br>&<br>Dr. Sarah A White, PhD<br>Statistician, Malawi-Liverpool-Wellcome Trust Clinical Research Programme, Blantyre, Malawi<br>Tel.: +265 1 676 444, FAX: +265 1 675 774<br>Mobile: +265 8 832 405, Email: swhite@mlw.medcol.mw<br>&<br>Dr. Yolande Coombes, PhD<br>Consultant, Blantyre Integrated Malaria Initiative<br>Tel.: +265 1 623 557, FAX: +265 1 623 557<br>Mobile: +265 8 828 125, Email: yolande@malawi.net<br>&<br>Dr. Elizabeth Molyneux, MD<br>Professor, College of Medicine, Dept. of Paediatrics<br>Tel.: +265 1 673 1198, FAX: +265 1 1 673 198<br>Mobile: +265 8 844 517, Email: emolyneux@malawi.net
Trial site:	Lungwena Training Health Centre<br>P.O.Box 230, Mangochi, Malawi
Laboratory analyses:	Lungwena Training Health Centre<br>P.O.Box 230, Mangochi, Malawi<br>&<br>UNC Project Laboratories<br>Lilongwe Central Hospital<br>Lilongwe, Malawi

1.2 Executive summary

Maternal anaemia, preterm deliveries and low birth weight are common in Sub-Saharan Africa and contribute significantly to the ill-health of pregnant women and infants. The present study is based on the assumption that these adverse outcomes can be prevented by improved antimicrobial management of malaria and sexually transmitted infections (STI) among pregnant women. To test the hypothesis, a randomised clinical trial following Good Clinical Practice (GCP) is planned to be carried out in Malawi, South-Eastern Africa.

A total of 1320 consenting women who present at a rural antenatal clinic after 14 but before 26 completed gestation weeks will be enrolled. One third of the women will receive antenatal care according to national recommendations, including regular visits to health centre, screening for pregnancy complications, haematinic and vitamin A supplementation and two doses of presumptive malaria treatment with sulfadoxine-pyrimethamine. Another third will receive otherwise the same care, but sulfadoxine-pyrimethmine treatment is given at monthly intervals. The final third receives standard antenatal care, sulfadoxine-pyrimethmine treatment at monthly intervals and two doses of presumptive STI treatment with azithromycin. Women are monitored throughout pregnancy and delivery and the newborns will be followed up for one year.

The primary outcome measure is proportion of preterm births in the three study groups. Secondary maternal outcomes include anaemia and malaria parasitaemia during pregnancy, at delivery and at 4 weeks after delivery, gestational weight gain and morbidity and STI prevalence after delivery. Secondary child outcomes consist of proportion of babies with low birth weight, mean birth weight, incidence of malnutrition during infancy, and mortality. Participant safety is systematically monitored throughout the intervention.

The study is planned to be started in June, 2003. Enrolment will take approximately 2.5 years and the total duration of the study is four years. The research team consists of Finnish and Malawian researchers and post-graduate students. Funding comes mainly from the Academy of Finland.

2. Background

2.1 Introduction

Pre-term birth, low birth weight and infant mortality are common in Malawi and many other countries from Sub-Saharan Africa. Recent community-based studies from Malawi and Mozambique suggest that up to 20% of all deliveries occur preterm (before 37 completed gestation weeks) (Schultz et al., 1994; Kulmala et al, 2000; Osman et al, 2001; N. van den Broek, personal communication). Approximately 15% of all newborns in Sub-Saharan Africa have a low birth weight (<2500 g) and 11% die before the age of one year (Unicef, 2001). Although the three entities are all multifactorial, they are also interrelated, i.e., pre-term births contribute to low birth weights, that in turn predispose the subjects for perinatal and infant death, often through malnutrition.

Several lines of evidence suggest that maternal malaria parasitaemia and sexually transmitted infections (STI) contribute to increased risk of maternal anaemia, preterm delivery and low birth weight (Meuris et al., 1993; Bloland et al., 1996; Schulman et al., 1999; Verhoef et al., 1999; Schulman et al., 2001). Among pregnant women enrolling for antenatal care in Malawi, the prevalence of malaria parasitemia and maternal anaemia has typically been reported at 20-45% and 49- 91 %, respectively (Steketee at al., 1996; Verhoef et al., 1999; Kulmala et al., 2000; Rogerson et al., 2000b). In the same areas, the prevalence of active syphilis has been approximately 10%, HIV 16-19%, gonorrhoea 8%, *Chlamydia trachomatis* -infection 6%, trichomoniasis 25% and bacterial vaginosis 15% (Kulmala et al, 2001; National AIDS Control Commission Malawi, 2001; Tsui et al., 2001; N. van den Broek, personal communication). Thus, STIs and malaria parasitaemia are both very common among pregnant women in Malawi, making an antimicrobial management strategy an apparently feasible option to prevent preterm birth and low birth weight.

2.2 Justification for making the study now

A wealth of data have documented improved malaria clearance among expectant mothers receiving two presumptive treatment doses of sulfadoxine-pyrimethamine during pregnancy (Schultz et al., 1994; Parise et al., 1998; Shulman et al., 1999). As a result, the national guidelines for malaria control in Malawi have since 1993 recommended this antimicrobial intervention; one treatment dose to be given at antenatal care enrolment and another one at 28-34 gestation weeks (Government of Malawi, 2002). Whereas this approach has undoubtedly been safe and beneficial, there have been a number of problems related to this approach, limiting the public health impact of the strategy.

The first problem related to the two-dose sulfadoxine-pyrimethamine treatment is poor customer compliance, possibly because it is difficult for the mothers to remember when they need the second dose (Verhoeff et al., 1998; Rogerson et al., 2000a; National Statistical Office (Malawi) and ORC Macro, 2001). Although suitable for DOTS, sulfadoxine-pyrimethamine is often prescribed to be taken at home, which further decreases compliance.

Additionally, the dosing interval appears too long, since one dose will be effective against malaria only for approximately four weeks (Sullivan et al., 1999). Finally, the emerging sulfadoxine-pyrimethamine resistance of *P. falciparum* strains threatens to make these antibiotics unsuitable for malaria treatment in Sub-Saharan Africa (Nzila et al., 2000; Sibley et al., 2001; Government of Malawi, 2002). New, more efficient therapeutic regimens are therefore needed.

Apart from the possible drug resistance, monthly dosing with sulfadoxine-pyrimethamine seems a logical choice for improved malaria control in pregnancy (Verhoeff et al. 1998). In Kenya, it was proven safe and more-effective than fewer-dose regimen and economic analysis suggested cost-effectiveness in populations with high HIV prevalence (Parise et al., 1998; Wolfe et al., 2001). Whereas other controlled trials have not been published, the World Health Organization already promotes this type of a frequent-dose intervention in the new guidelines for managing malaria in pregnancy (Dr. B. Nahlen, personal communication).

Considering the frequency of STIs, their known association with preterm birth and low birth weight and the potential of antimicrobial treatment to prevent these outcomes when targeted to infected individuals (Hauth et al., 1995; Regan et al., 1996; Lamont, 1999), presumptive STI-treatment for all pregnant women provides another feasible antenatal intervention in Sub-Saharan Africa. However, only a few trials have been reported. In Kenya (n=400), a single intramuscular dose of ceftriaxone reduced the prevalence of gonorrhoea from 4.2% to 1.8%, postpartum endometritis from 10.4% to 3.8%, and low birth weight rate from 9.2% to 4.0% (Temmerman et al., 1995). In Uganda (n=4036), a single-time oral treatment consisting of 1 g azithromycin, 400 mg cefixime, and 2 g metronidazole cleared various STIs and reduced the incidence of preterm delivery by 23%, low birth weight by 32% and neonatal mortality by 17%, as compared to placebo treatment (Gray et al., 2001). No major adverse reactions were reported from either study.

The optimal dose of azithromycin in the treatment of STIs is unknown, as no direct dose comparison studies have been conducted. Most STIs can be treated with a single 1 g oral dose, but some studies have questioned its adequacy in the treatment of gonorrhoea, because of which the U.S. guidelines recommend a 2 g dose for this illness (Centers for Disease Control and Prevention, 2002). Many other studies, however, have documented a favourable result also with 1 g treatment (Odugbemi et al., 1993; Waugh 1993; Gruber at al., 1997; Swanston et al., 2001) and in Europe this lower dose is indicated also for gonorrhoea (Pfizer Pharmaceuticals Group, 2002). Adverse effects (mainly abdominal) are less common with the lower 1 g dose (Pfizer Inc., 2002).

If presumptive treatment of STIs is considered during pregnancy, azithromycin antibiotic has several advantages. It has a broad antimicrobial spectrum, covering e.g. gonorrhoea, chlamydia, chancroid, and possibly syphilis. It is safe in pregnancy and can be administered under direct observation as a single oral dose or as two doses, one at the beginning of antenatal care and one later during pregnancy (Gray et al. 2001; Centers for Disease Control and Prevention, 2002). Finally, it also has antimalarial activity (Anderson et al., 1995; Taylor et al., 1999, Ohrt et al., 2002), which may become important in Sub-Saharan Africa if parasite
12 resistance against sulfadoxine-pyrimethamine continues to increase. Currently, the Kenya Medical Research Institute is conducting a phase II study to determine the antimalarial effects of azithromycin in combination with sulfadoxine-trimethoprime or with chloroquine in semi-immune pregnant women (Monica Parise, personal communication).

In the light of above, it is justified to implement a clinical trial, testing the health effects of presumptive antimalarial treatment of pregnant women in Sub-Saharan Africa with monthly doses of sulfadoxine-pyrimethamine, either alone or in combination with presumptive STI treatment with azithromycin.

2.3 Objectives of the study

In broad terms, the current study is set up to test the hypothesis that in rural Malawi maternal health could be improved and the prevalence of pre-term births and low birth weight reduced by improved management of maternal malaria and sexually transmitted illnesses during pregnancy.

The specific objectives include detailed analysis of the health effects of two different antenatal interventions (monthly sulfadoxine-pyrimethamine therapy and monthly sulfadoxine-pyrimethamine in combination with occasional azithromycin treatment). The analyses will focus on the following issues:

a. The effect of antenatal interventions on the duration of pregancy, birth size and perinatal and neonatal mortality
b. The effect of antenatal interventions on maternal weight gain and anaemia during pregnancy
c. The effect of antenatal interventions on the prevalence of maternal malaria infection and febrile illnesses during pregnancy
d. The effect of antenatal interventions on the incidence of moderate (WAZ <-2) underweight during infancy
e. The effect of antenatal interventions on infant mortality
f. The economic and cultural feasibility of the tested interventions
g. The development of fundal height and foetal measurements (ultrasound assessed) in relation to the duration of pregnancy
i. The value of fundal height measurement and Ballard score analysis in the assessment of the duration of gestation (a comparison to ultrasound assessment).
j. The prevalence and classification of main delivery complications
k. The occurence of antimicrobial resistance against sulfadoxine-pyrimethamine in malaria-parasites found in the study area

3. Methods for the study

3.1 General design

A phase 3, randomised, parallel-group investigator-blinded clinical trial

3.2 Place of research

The study will be carried out in Lungwena, Mangochi District, Southern Malawi, where the current study group has had an ongoing research project on maternal and child health since 1994. The area is rural, approximately 100 km^2 in size and the nearest town (Mangochi) lies some 30 km away. The population is about 20 000, most of whom are subsistence farmers, Muslim and belong to the Yao tribe. The Yaos are typically organised matrilineally, which means that women and children reside near the extended maternal family, whereas men move to marry. The general educational level in Lungwena is very low (only 41% of men and 14% of women are literate).

A government health centre, which also serves as a centre for a community health teaching and some primary health care interventions, is located in the middle of the area. This facility is equipped with solar electricity, research office and personnel, and a basic laboratory. It provides the local population with free preventive and curative modern health services such as family planning, antenatal and delivery care, and treatment of common illnesses.

3.3 Study population

3.3.1 Participants

The crude birth rate in the study area is approximately 45 births / 1000 inhabitants. Almost all pregnant women attend the antenatal clinic at the health centre several times during the pregnancy (Kulmala et al., 2000). The study population will be drawn from those pregnant women, who present at the health centre before they have completed their 26th gestation week.

The enrolment criteria for study participants will be the following:

Inclusion criteria:

- -signed informed consent
- -age 15.00 years or older
- -ultrasound confirmed pregnancy
- -mother has felt the movements of the foetus (quickening)
- -foetal age of at least 14 but not more than 26 completed gestation weeks (based on ultrasound assessment)
- -maternal availability for follow-up during the entire period of the study

Exclusion criteria:

- -known maternal tuberculosis, diabetes, kidney disease, or liver disease
- -any severe acute illness warranting hospital referral (judged by the attending nurse-midwife)
- -mental disorder that may affect comprehension of the study or success of follow-up
- -twin pregnancy
- -pregnancy complications evident at enrollment visit (moderate to severe oedema, blood Hb concentration < 5 g / dl, systolic blood pressure (BP) > 160 mmHg or diastolic BP > 100 mmHg)
- -prior receipt of azithromycin during this pregnancy
- -receipt of sulfadoxine and pyrimethamine within 28 days before enrollment
- -known allergy to drugs containing sulphonamines, macrolides or pyrimethamine
- -history of anaphylaxis
- -history of any serious allergic reaction to any substance, requiring emergency medical care
- -concurrent participation in any other clinical trial

3.3.2 Study interventions

The study will compare the effect of two more active infection management strategies to that of the standard antenatal care.

The *control group* will receive the standard Malawian antenatal care including:

- -regular antenatal (ANC) visits at 4 week intervals until 36 completed gestation weeks and then at one-week intervals until delivery.
- -screening for syphilis with VDRL-test. If test positive, treatment of the mother, her sexual partner and the newborn baby with intramuscular injections of benzthatine penicillin (adult dose 2.4 mU, newborn dose 50 kU / kg)
- -gestational iron (ferrous sulphate, 200 mg daily) and folic acid (5 mg daily) supplementation starting at first antenatal visit and continuing until delivery.
- -presumptive intermittent malaria treatment with sulfadoxine – pyrimethamine twice during pregnancy; at enrollment visit and between 28th and 34th week of gestation. Each treatment dose contains: tbl sulfadoxine-pyrimethamine, 3 tablets orally, each containing 500mg of sulfadoxine and 25mg of pyrimethamine.
- -two treatments with azithromycin-like placebo: at enrollment visit and between 28th and 34th week of gestation. Each treatment dose contains: placebo tbl looking like Zithromax® tbl, 2 tablets orally
- -vitamin A supplementation (200 000 IU oral capsule) immediately after delivery and at 6 months post-delivery
- -treatment of other maternal illnesses, pregnancy complications, infant malnutrition and other conditions as medically indicated. In case of malaria, treatment with quinine (tbl Quinine® 300 mg, 2 tablets orally three times a day for 5 days).

- -in addition to the standard ANC care, the control group is offered voluntary counseled testing for human immunodeficiency virus infection. If test positive, treatment of the mother at the onset of delivery and the newborn within 72 h of delivery with nevirapine. Adult dose: tbl Viramune®, 1 tablet orally, containing 200 mg nevirapine. Newborn dose: suspension Viramune®, 3.5 ml orally, containing 7

mg nevirapine (newborn dose approximately 2 mg / kg).

The *intervention group A* will receive more active presumptive treatment for malaria:
-standard antenatal and other health care as indicated above, including VDRL and HIV –testing and iron, folate and vitamin A supplementation, and appropriate medical treatment of health problems.
-presumptive intermittent malaria treatment with sulfadoxine – pyrimethamine at 4 week intervals (more frequent dosing), starting from the enrolment visit and finishing latest at 37 completed gestation weeks. Each treatment dose contains: tbl sulfadoxine-pyrimethamine, 3 tablets orally, each containing 500mg of sulfadoxine and 25mg of pyrimethamine.
-two treatments with azithromycin-like placebo: at enrollment visit and between 28th and 34th week of gestation. Each treatment dose contains: placebo tbl looking like Zithromax® tbl, 2 tablets orally

The *intervention group B* will receive more active presumptive treatment for malaria and sexually transmitted illnesses:
-standard antenatal and other health care as indicated above, including VDRL and HIV –testing and iron, folate and vitamin A supplementation, and appropriate medical treatment of health problems.
-presumptive intermittent malaria treatment with sulfadoxine – pyrimethamine at 4 week intervals (more frequent dosing), starting from the enrolment visit and finishing latest at 37 completed gestation weeks. Each treatment dose contains: tbl sulfadoxine-pyrimethamine, 3 tablets orally, each containing 500mg of sulfadoxine and 25mg of pyrimethamine.
-two presumptive treatments for sexually transmitted infections with azithromycin: at enrollment visit and between 28th and 34th week of gestation. Each treatment dose contains: tbl Zithromax®, 2 tablets orally, each containing 500mg of azithromycin.

The study drugs are given under direct observation, together with fruit juice and bisquits.

All participants will be informed about the benefits of pyrethrine-impregnated bet-nets and encouraged to procure them for their own and their children's use.

3.4 Study period

In total, the data collection will run for 4-4.5 yars. The detailed time schedule is below.

August to December 2002	Finalising research plan<br>Approval by the ethics committee (COMRC)
January to May 2003	Consultation with community leaders<br>Staff recruitment and training<br>Developing practical arrangements and SOPs
June 2003 to October 2005	Enrolment

July 2004	Interim analysis of safety
October 2005 – May 2006	Data collection, maternal / newborn outcome
June 2006 – May 2007	Data collection, infant outcome<br>Data analysis on maternal / newborn outcome<br>Results dissemination on analysed data
June 2007 – December 2007	Data analysis on infant outcome<br>Results dissemination on analysed data

3.5 Sample size, its justification, and recruitement rate

3.5.1 Sample size

The selected sample size is 440 women per group (1320 in total), calculated from the expected numbers of pre-term deliveries (primary outcome).

Based on our own earlier results and those of others in Malawi and Mozambique, 20% of all deliveries are at present estimated to occur before 37 completed gestation weeks (Schultz et al., 1996; Kulmala et al., 2000; Osman et al., 2001; N. van den Broek, personal communication). Due to a possible increase in the use of bed-nets, this proportion is estimated to be 18% in the control group for the present study. A 40% decrease in the intervention group (from 18% to 10.8%) is considered clinically significant and achievable. A sample size of 400 per group will produce an 80 % power and 95 % confidence to detect such a difference between the intervention and the control groups. Because of an estimated 10% loss to follow-up (see 3.14), 440 women are enrolled to each group.

For secondary outcomes, the chosen sample size provides 80% power and 95% confidence to detect a 0.5 gestation week difference in the mean duration of pregnancy, 95 g difference in mean birth weight and a drop in the incidence of low-birth weight from 20% to 12.5%.

3.5.2 Planned recruitment rate

The planned recruitment rate is 10 women / week or 535 women / year, i.e. the recruitment will take approximately 2.5 years. This is estimated from the number of people living in Lungwena Health Centre catchment area (20 000) and a crude birth rate of approximately 45 births / 1000 inhabitants / year. Of the annual 900 pregnant women, 70% are estimated to begin antenatal care at the health centre before 26 completed gestation weeks and 85% of them are assumed to enroll in the study.

The estimated enrolment rates at health centre antenatal clinic and the clinical trial are based on an earlier cohort study and health centre statistics in the same area, where 60-70% of all pregnant women presented at the health centre before 26 completed gestation weeks and 99% of them enrolled in the offered study (Kulmala et al., 2000). For the present trial, the timely enrolment at health centre is estimated at least as high as before (due to community sensitization) and enrolment a bit lower than the earlier study (due to more intensive follow-up).

3.6 Data collection

3.6.1 Recruitment

All pregnant women attending the antenatal clinic at Lungwena Health Centre are approached and briefed about the study (Appendix 1). Women wishing to participate and signing an informed consent form (Appendix 2) will undergo an interview on socio-economic background and health history (Appendix 3-4), a medical examination (Appendix 5), an ultrasound assessment for duration of pregnancy (Appendix 6) and selected laboratory analyses (Appendix 7). Those meeting the predefined criteria will be enrolled and randomly allocated to different study groups (Appendix 8-9).

Before enrolment, the purpose and methodology of the study will be discussed with community leaders and in larger village meetings. On these occasions, the importance of early antenatal care enrollment is emphasized.

3.6.2 Outcome measures and safety analysis

Primary outcome for efficacy:

-percentage of pre-term deliveries (birth before 37 completed gest. weeks)

Secondary outcomes, maternal health:

-mean maternal blood haemoglobin concentration (Hb) during pregnancy (separately for each antenatal visit and for the visit at 4 weeks after delivery)

-percentage of women with mild, moderate or severe anaemia (Hb < 11, 8, and 5 g/dl, respectively) at every antenatal visit and at 4 weeks after delivery

-percentage of women with peripheral blood malaria parasitemia and mean parasite density (at first visit, at approx.32 gestational weeks and at delivery (only for those delivering at Lungwena Health Centre)

-percentage of women with cord blood or placental malaria parasitemia and mean parasite density at delivery (only for those delivering at Lungwena Health Centre)

-maternal weight gain during pregnancy

-mean number of illness days during pregnancy (self-reported symptoms of malaria and other illnesses).

Secondary outcomes, child health:

-percentage of low birth weight babies (< 2500g)

-mean birth weight

-mean duration of gestation

-percentage of low chest circumference (< 30cm, indicator of small birth size)

-percentage of low chest or head circumference (chest < 30 cm, head < 31 cm)

-incidence of moderate (WAZ <-2) underweight during infancy

-perinatal, neonatal and infant mortality (between 0 and 6 months of age, after 6 months of age malnourished infants may enroll in nutrition rehabilitation programmes)

Primary outcome for safety

-number of serious and any adverse reactions (especially rash, vomiting, abdominal cramps, diarrhoea)

3.6.3 Frequency and duration of follow-up

Figure 1 shows the outline of the recruitment, group allocation and follow-up. In brief, all participants are seen at the health centre at 4-week intervals until 36 completed gestation weeks and weekly thereafter. At each visit, the mothers will undergo an interview, a routine antenatal investigation (Appendix 10) and blood sampling (normally 250 μl from finger-tip, at first visit 5 ml from median antebrachial vein). Pelvic ultrasound (assessing only the foetal biparietal diameter, length of the femur and pulsation of the heart, but not foetal morphology) is performed for everybody at first visit and for a subgroup of 300 women (100 both interventions, 100 control) at each visit. If the fetus is found dead (no heart pulsation), the mother is referred to Mangochi District Hospital for appropriate management). All women will be offered screening for syphilis and HIV-infection at first ANC visit.

Within 48 hours of delivery, a research assistant will make a home-visit, during which she will interview the mother and (if possible) the person attending the delivery about delivery events (Appendix 11-12) and measure the birth measurements, assess the duration of pregnancy by modified Ballard score (Appendix 13). At four weeks after delivery, the mother and the child will undergo a medical examination at the health centre (Appendix 14). For the assessment of growth and mortality in infancy, the babies are examined at 3, 6, 9, and 12 months of age at the health centre (Appendix 15).

From the blood samples taken at each visit, 100 μl is stored on filter papers (2 spots, each 50 μl). These samples will be used to produce descriptive data on the incidence and prevalence of sulfadoxine-pyrimethamine resistant malaria parasites in the study area.

A 10 ml urine sample will be collected from the partipating women at 4 weeks after delivery. These samples are stored at –20°C, and used within a month of sample collection to determine the impact of the intervention on the prevalence of *C. trachomatis* or *N. gonorrhoae* infection. Women who are found infected (and their sexual partners) are offered appropriate antibiotic therapy (250 mg siprofloxasin orally as a single dose for gonorrhoea, 1 g azithromycin orally as a single dose for chlamydia).

Women, who were treated for confirmed syphilis at enrolment (a positive TPPA test) will be offered a possibility to have a follow-up blood test taken at 4 weeks after delivery. Additionally, they are offered to have their children tested for syphilis at 6 months of age (when passively transferred maternal antibodies have disappeared). All women and children found infected will be treated appropriately with antibiotics.

Figure 1. Outline of the follow-up of LAIS participants

Screening, recruitment, and group allocation

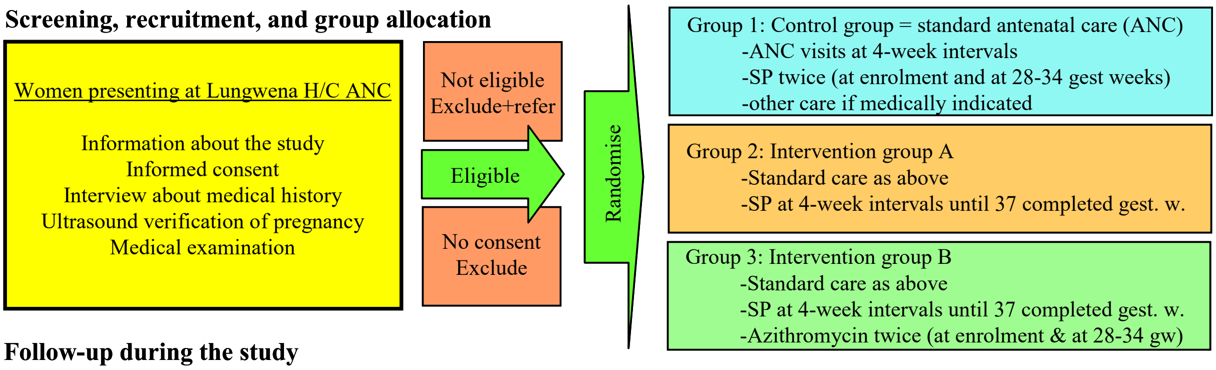

Follow-up during the study

Data on outome measures			Duration of pregnancy (completed gestation weeks)											Duration of infant follow-up				
**Variable**	**Location**	**Analyser**	**14 - 17**	**18 - 21**	**22 - 25**	**26 - 29**	**30 - 33**	**34 - 36**	**37**	**38**	**39**	**40**	**Delivery**	**4 wk**	**3 mo**	**6 mo**	**9 mo**	**12 mo**
Duration of pregnancy	Home	Res ass.											⇑					
GC and Chlamydia preval.	H Centre	Lab ass.												⇑				
Mat. blood haemoglobin[1]	H Centre	Lab ass.	⇑	⇑	⇑	⇑	⇑	⇑	⇑	⇑	⇑	⇑	(⇑)	⇑				
Mat.malaria parasitaemia[1]	H Centre	Lab ass.	⇑				⇑						(⇑)					
Maternal weight gain	H Centre	Res nurse	⇑	⇑	⇑	⇑	⇑	⇑	⇑	⇑	⇑	⇑	⇑	⇑				
Maternal illness days	H Centre	Res nurse	⇑	⇑	⇑	⇑	⇑	⇑	⇑	⇑	⇑	⇑						
Birth size of babies	H Centre	Res ass.											⇑					
Infant growth	H Centre	Res ass.												⇑	⇑	⇑	⇑	⇑
Infant mortality	H Centre	Res ass.												⇑	⇑	⇑	⇑	⇑
Side effects	H Centre	Res nurse		⇑	⇑	⇑	⇑	⇑	⇑	⇑	⇑	⇑		⇑				

[1] *Measured at delivery only from those, who give birth at Lungwena Health Centre. At delivery, malaria parasitaemia measured from peripheral blood, cord blood and the placenta.*

Other collected data			Duration of pregnancy (completed gestation weeks)											Duration of infant follow-up				
**Variable**	**Location**	**Analyser**	**14 - 17**	**18 - 21**	**22 - 25**	**26 - 29**	**30 - 33**	**34 - 36**	**37**	**38**	**39**	**40**	**Delivery**	**4 wk**	**3 mo**	**6 mo**	**9 mo**	**12 mo**
Background information	H Centre	Res ass.	⇑															
Routine antenatal care[2]	H Centre	HC nurse	⇑	⇑	⇑	⇑	⇑	⇑	⇑	⇑	⇑	⇑						
Pelvic ultrasound[1]	H Centre	Res nurse	⇑	(⇑)	(⇑)	(⇑)	(⇑)	(⇑)	(⇑)	(⇑)	(⇑)	(⇑)						
Matern. fundal height gain	H Centre	HC nurse	⇑	⇑	⇑	⇑	⇑	⇑	⇑	⇑	⇑	⇑						
Delivery complications	Home	Res ass.											⇑					
Maternal HIV-infection	Blantyre	Collabor.	⇑															
Drug resistance in *P falc.*	Blantyre	Collabor.	⇑															
Maternal syphilis (VDRL)	H Centre	Lab ass.	⇑											(⇑)				

[1] *After the enrolment, measured only from a subgroup of mothers (100 in intervention + 100 in control groups).* [2] *Routine antental care includes an interview for recent medical history, measurements of weight and of blood pressure, inspection for oedema, measurement of urine protein if indicated by other findings, auscultation for foetal heart sounds, external abdominal palpation and measurement of fundal height.*

3.6.4 Methods used for data collection

Primary efficacy outcome: The duration of pregnancy at birth will be determined based on ultrasound assesment (by a research nurse) of foetal bi-parietal diameter and femur length at first antenatal visit.

Secondary maternal outcomes: Blood haemoglobin concentration is measured with a Hemo-Cue® –instrument from a finger prick sample (on first visit from venous blood). At selected ANC visits, the same blood sample is used for the assement of peripheral blood malaria parasitaemia (Giemsa -stained thick and thin blood films). For women delivering at Lungwena health centre, malaria parasitaemia will be assessed also from peripheral and cord blood and the placenta.

Maternal weights are measured by a research assistant with a digital bathroom scale (reading increment 200 g).

Maternal morbidity is determined by 4-weekly interviews about the number of days, when the mother has had symptoms of malaria or other illnesses.

The prevalence of maternal *N. gonorrhoeae and C. trachomatis* infections will be dermined with a DNA-amplification -method from urine samples, taken at the 4-week post-delivery

visit and stored at –20ºC for a maximum of one month.

Secondary child outcomes: Birth weight, length, and chest and head circumpherence will be measured within 48 hours of delivery by research assistants, using spring scales (100 g reading increment), self-made length boards (5 mm reading increment) and elastic tapes (1 mm reading increment).

Child growth is monitored by quarterly measurement (at the health centre) of infant weight (electronic scale, recorded to the nearest 10 g), length (infantometer, recorded to the nearest 1 mm) and head circumference (elastic tapes, recorded to the nearest 1 mm).

Child survival is queried from the guardians at their quarterly visits to the health centre.

Safety outcomes: Adverse reactions to study medications are queried with structured forms (Appendix 16) at every ANC visit and at any other visits of the mother to the health centre.

Data from all non-scheduled visits to the health centre, either at antenatal clinic or at out-patient department, are documented in structured forms (Appendix 17)

Other collected data: Socioeconomic and demographic background of the mother and her medical history are queried with a structured interview at enrolment visit. Routine antenatal assessment will include an interview about recent medical history, measurements of weight and blood pressure (with an electronic sphyghomanometer), inspection for oedema, measurement of urine protein (chemical screening with dipsticks) if indicated by other findings (high / increasing blood pressure or oedema), auscultation for foetal heart sounds, external abdominal palpation and measurement of fundal height (with elastic tapes, reading increment 1 mm). Pelvic ultrasound will be done with a portable analyser by a research nurse.

Delivery complications will be queried with structured forms from both the mother and the person attending the delivery (if possible).

For comparison with the ultrasound-obtained data, the duration of gestation will be calculated from fundal height at first antenatal visit (measured by a research nurse) and the modified Ballard score within 48 hours of birth (by a research assistant, Verhoeff et al., 1997).

Maternal HIV status is determined (after counseling and informed consent) at enrolment from separated sera with two independent antibody-ELISAs (Determine, Abbot Laboratories, Abbot Park, USA and Uni-Gold, Trinity Biotech plc, Bray, Ireland). The same serum samples are used for syphilis screening (VDRL carbon antigen, Biotech Laboratories, Ipswich, UK). All VDRL positive women will receive penicillin treatment, but the screening results are later confirmed with a TPPA-assay (Serodia-TPPA, Fujirebio, Tokyo, Japan). The prevalence and pattern of sulfadoxine-pyrimethamine resistance in *P. falciparum* strains is assessed with DNA technology from filter-paper impregnated blood samples, taken from malaria-positive individuals at various ANC visit.

Bed-net use (frequency of use, age of net and its impregnation history) will be queried and documented at each ANC visit and at 4 weeks after delivery.

3.6.5 Practical arrangements for allocating participants to groups

The study participants will be randomised (with computer-generated random number list) in blocks of 9-18 into control and intervention groups. From all groups, 100 individuals will further be randomised into a more intensive ultrasound follow-up. The randomisation list will be placed in three separate sealed opaque envelops, to be stored by the principal investigator (University of Tampere, Finland), a member of the review board (Blantyre, Malawi), and a co-ordinating investigator at the study site (Lungwena Training Health Centre).

Based on the randomisation list, individual envelopes will be made, marked on top with an individual identification number and containing a paper indicating the appropriate group allocation. When a new participant is enrolled, a research assistant picks the lowest available randomisation number and opens the respective sealed and opaque envelope. The paper included in the envelope will indicate the group allocation. The office assistant will keep a register of identification codes and respective group allocations of all enrolled participants, but this register will not be shown to any people responsible for outcome evaluation.

3.6.6 Methods for protecting against other sources of bias

Randomisation, group allocation, and distribution of study drugs will be done by a research assistant not participating in the outcome evaluation. The researchers and the staff measuring the outcome variables will remain blinded to the group allocation until the end of data collection.

3.6.7 Likely rate of loss to follow-up and participant tracing

Ten per cent of the women and their offspring are expected to be lost to follow-up. This is a conservative estimate, based on the potential problems mentioned in the previous paragraph (3.14) and the fact, that the corresponding rate was 4.8% in an earlier study from the same area (Kulmala et al., 2000).

If a pregnant woman misses a planned visit with more than 4 weeks, the study co-ordinator will send a research assistant to make a home visit and see if the baby has already been born. If so, the research assistant interviews the mother and delivery attendant about delivery events, takes the antropometric measurements from the newborn and post-natal follow-up will continue as planned. If not, the mother is reminded about the trial and the importance of follow-up visits. If the participant still fails to attend the ANC, the reminder home visits are repeaeted at 4-week intervals until delivery.

If an infant misses a planned visit with more than 2 weeks, the study co-ordinator will send a research assistant or other member of the research team to make a home visit and remind the guardian about the trial and the importance of follow-up visits. If the participant still fails to attend the H/C within the next 2 weeks, the study team will make another home visit and interview the guardian and measure the child's growth at home.

3.6.8 Management of the study

Day-to-day management. The study team on the research site will have a three-member core group (a coordinating investigator, a study nurse, an office assistant), responsible for smooth implementation of the study. The core group will meet weekly, to review progress of enrolment and follow-up, to discuss any problems encountered and to accept a general plan of activities for the subsequent week.

An office assistant will keep records and produce weekly graphic summaries of the success of enrolment and follow-up. She will also create a master chart of visits for the enrolled participants. Based on this, she will make a weekly duty plan for the other members of the team. The office assistant will distribute these plans to members of the study team at the end of each week. Once a month, the study team will have a larger staff meeting to discuss the progress and any acute issues related to the study.

A systematic participant flow will be designed for the health centre, ensuring smooth antenatal visits for the participants. The participants will report to the office assistant, get all necessary forms for source data, visit appropriate study stations (information about the study, background interview, antenatal examination, ultrasound, HIV-counseling, laboratory) and finally return to the office assistant, give her the source data, obtain planned drug treatment and agree on the date of the subsequent appointment. The office assistant but no other members of the study team will know the group allocation of each participant.

At each study station, research assistants will document their findings on the source data forms carried by the participant. Additionally, the research assistants will keep log-books of all participant contacts at their study stations.

The intervention drugs needed in the trial are either provided by the manufacturer (Zithromax®) or purchased from the Malawi Central Medical Stores (sulfadoxine-pyrimethamine). Other drugs needed in the antenatal clinic are purchased from the Central Medical Stores or obtained from a drug donation program (Viramune®). The drugs are prepacked for individual participants and visits (each participant will have a separate drug envelope, containing 7 smaller envelopes with study drugs for each individual visit), stored in safe containers and appropriate conditions and accounted for by the study office assistant.

3.6.9 Scientists and their responsibilities

Dr. Per Ashorn, MD is a paediatrician and paediatric infectious disease specialist who currently holds a position as a Senior Scientist at the University of Tampere, Finland. Between 1993 and 1995, Dr. Ashorn worked as a Medical Officer and as a Senior Clinical Lecturer in Malawi and since then he has coordinated studies on maternal and child health at the proposed study site. Dr. Ashorn is one of the two principal investigators of the present study and he coordinates its planning and analysis and bears the overall responsibility for its implementation and financing.

Dr. Chiwoza Bandawe is the head of Community Health Department, College of Medicine, Malawi (COM). He provides on-site supervision for the PhD students involved in the study, especially in relation to behavioural and cultural questions.

Dr. Teija Kulmala, MD is a medical doctor who works as a Medical Advisor for a Finnish NGO, the Family Federation of Finland. She did her doctoral thesis on maternal health in Malawi, and she has analysed much of the data used in the planning of the present study. Dr Kulmala is a co-principal investigator and she participates in the design, implementation and analysis of the study.

Dr. Mari Luntamo, MD is a general practitioner who will act as a field coordinator of the proposed study for the first one and half years. Her responsibilities include the practical implementation and data analysis of the study with the support of the study group. During the

study, Dr. Luntamo will check all data, verify them and complete the case report forms. The work will form Dr Luntamo's postgraduate studies (Doctorate in Medical Science at the University of Tampere, Finland) and she will be the responsible author of the first block of the eventual publications.

Dr Kenneth Maleta, MBBS, is a medical doctor, who has specialised in rural health problems and who will complete his doctoral studies (at the University of Tampere, Finland) before the onset of the proposed study. Dr. Maleta has a position as assistant lecturer in Community Health at the College of Medicine, University of Malawi and he will act as a local supervisor for the field-coordinators of the study.

Dr. Bernard Mbewe is the vice-head of Community Health Department (COM). He will coordinate the study after Dr Luntamo's field work. His/her responsibilities will be the same as those of Dr Luntamo. The work will form his postgraduate studies (Doctorate in Medical Science at the University of Tampere, Finland) and he will be the responsible author of the second block of the eventual publications.

3.6.10 Responsibilities of the research assistants

The team will consist of one study nurse/midwife, three office assistants, one laboratory assistant and three field assistants. Additionally, two nurse/midwives working at the Lungwena Training Health Centre will work part time for the study. Apart from one of health centre nurses, all members of the team have several years of research experience from the proposed site.

The study nurse/midwife will act as a chief of staff and provide the participants with information about the study, counsel them about HIV before and after antibody testing and do the ultrasound assessments. One office assistant will allocate the participants into treatment groups, co-ordinate the study visits, collect, record and store all source data, and distribute the study drugs. The other office assistants and the field assistants will interview the mothers, collect information on the participants' background and delivery events and measure the birth size and growth of the babies. The health centre nurse mid-wives will be responsible for the antenatal medical examinations.

3.7 Data management and analysis

3.7.1 Data handling and record keeping

Written records (in the forms of structured questionnaires) will be made from each data-producing contact between the study participants and the study team. These source data are identified (each sheet containing date, participant number and signature of the data collector) and stored in individual participant folders.

Based on the source data, Case Report Forms (CRF) will be provided for each subject. All data on the CRF will be legibly recorded in ink. Any corrections will be made by striking through the incorrect entry with a single line and entering the correct information adjacent to it. The corrections will be initialled and dated by the investigator or a designated, qualified individual. Any requested information that is not obtained as specified in the protocol or changes in medication will have an explanation noted on the CRF.

All Case Report Forms will be filled out by an office assistant, then reviewed and signed by an investigator to indicate their correctness.

All documentation regarding the participants, including the laboratory samples, source data and the Case Report Forms, will be identified with appropriate participant codes, both on paper and in computer files. The names will only appear on informed consent forms and a separate coding list.

The investigators shall maintain the records of disposition of drug receipts and drug inventory logs and their copies of the Case Report Forms and regulatory documents (informed consents, ethical approval) for 15 years after the end of the study. All records will be kept in a secure place. Clinical information will not be released without written permission of the subject, except as necessary for monitoring.

No external data monitoring is pre-planned for the study. The quality assurance of LAIS is done through the use of detailed standard operating procedures (SOP) and weekly internal monitoring rounds. The investigators will make study documents (e.g., consent forms, drug distribution forms, case report forms) and pertinent hospital or clinic records readily available for inspection only to those national or international authorities who have a legal right to monitor proper conduct of clinical trials.

3.7.2 Analysis strategy

The analysis will compare the preselected outcomes (chapter 3.6.2) in the intervention and control groups. All women and children will be analysed in the group where they were initially randomised, i.e. on an intention to treat –basis. For most outcomes, the analysis will compare group means or proportions at a single time-point and results are expressed as absolute differences and their 95% confidence intervals. The statistical significance of observed group differences is calculated with chi-square test (categorical variables), Student's t-test (quantitative variables with normal distribution) or with a rank score test (quantitative variables with non-normal distribution). For secondary outcomes, Bonferroni corrections will be made before the interpretation of calculated p-values. For single-time point analyses, individuals lost to follow up (with no outcome data) will be excluded.

Comparison of infant malnutrition and mortality in the three groups will also be done with a survival analysis, i.e. using a time-dependent outcome variable. All enrolled participants are included in these analyses.

3.7.3 Frequency of analyses

The randomisation code is broken (but not given to research assistants measuring the growth of infants) after the last woman and child have attended the 4 week follow-up visit, i.e. data on pregnancy and puerperal period are complete. The main analysis will be done at this point. The analysis of infant growth will be done a year later, when all children have become one year old.

Besides the above efficacy assessment, one interim analysis for safety is planned to take place when 500 women have been enrolled to the trial and followed up for at least 10 weeks during pregnancy. For this analysis, the review board will be provided with numbers and types of adverse effects in the three groups. The interim analysis will not, however, look at the actual

treatment of the groups (which is control or active intervention), unless the panel requests so based on the interim results.

3.7.4 Subgroup analyses

Three subgroup assessments are planned in advance. These include analyses stratified either by maternal HIV-status (seropositive / seronegative / not known) or by maternal parity (primipara / secundipara / multipara). Additionally, a subgroup analysis is planned for those, who were enrolled before 24 completed gestation weeks (because ultrasound assessment of gestational age is believed to be most reliable before this cut-off).

3.7.5 Co-enrollment guidelines

Infants who become malnourished (WAZ<-2) are withdrawn from the follow-up and they may after 6 months of age be enrolled into intervention trials for malnutrition rehabilitation.

3.7.6 Co-ordination with other studies

The study involves collaboration with Dr. Irving Hoffman (University of North Carolina) and the UNC Project Laboratories, Lilongwe Central Hospital, Malawi. Dr. Hoffman will be responsible for the *C. trachomatis* and *N. gonorrhoea* –testing from urine samples collected as part of the proposed study. Additional collaboration involves Dr Steven Meshnick (University of North Carolina) and Dr. Stephen Rogerson (University of Melbourne), who are coordinating a malaria research project elsewhere in Malawi (Blantyre). Drs Meshnick and Rogerson will co-ordinate the analysis on malaria-parasite resistance against sulfadoxine-pyrimethamine (using samples from Lungwena).

4. Presentation, dissemination and use of the results

The results will be distributed and discussed with the local community and representatives of the Ministry of Health and Population and College of Medicine, Malawi. Main findings will be published in international peer-reviewed journals and the Malawi Medical Journal.

The results can be used in the planning and development of antenatal care in Malawi and other countries with endemic malaria and high frequency of preterm deliveries. They may have policy implications for the management of pregnant women at Malawian health centres.

The study material will also be used for post-graduate training of Malawian and Finnish students.

5. Ethical considerations

5.1 Informed consent and compensation to participants

All potential participants will receive structured information about the study during their antenatal clinic visit (Appendix 1). Those interested in participation, will be invited to a private discussion with study personnel, during which the potential participants may ask questions about the study. Those wishing to participate will then sign an informed consent form, indicating the voluntary nature of the study and the participants' right to discontinue follow-up at any point (Appendix 2).

The participants are not paid for enrolment or follow-up, but they will be compensated for their time with a bar of soap at enrolment, at 32-week antenatal visit and after delivery. They will also be reimbursed with 50 kwacha (1 USD) for informing the study team rapidly about the delivery, when it has taken place. All visits and study medications will be free of charge to the participants and the study team will support study babies' nutritional status by giving them a package of likhuni phala (Maize / soy flour) at 6 and 12 months of age.

5.2 Possible risks to the safety of participants involved in the study

Both drugs used in the study are registered and they have proven safe for use in pregnancy under conditions similar to the intended trial (Parise et al., 1998; Shulman et al., 1999; Gray et al., 2001). Two doses of SP as a presumptive intermittent treatment for malaria has been the national standard in Malawi since 1993 (Government of Malawi, 2002) and no problems have been noticed in 3 studies (a total of 2533 subjects) reporting a more frequent dosing (Parise et al. 1998, Verhoeff et al. 1998, Shulman et al. 1999). Azithromycin is recommended by the Centers for Disease Control and Prevention (USA) as an alternative regimen for the treatment of several different sexually transmitted infections among pregnant women (Centers for Disease Control and Prevention 2002).

Despite the above, there are theoretical risks related to the administration of two separate drugs to pregnant women. Both sulfadoxine-pyrimethamine and azithromycin (Zithromax®) have been associated with occasional adverse effects; SP most commonly with blood dyscrasias and various allergic, gastrointestinal, central nervous system and respiratory reactions (Roche Pharmaceuticals, 2002) and Zithromax® with mild gastrointestinal symptoms and, less commonly, cardiovascular, genitourinary, nervous system and allergic reactions have been reported (Pfizer Inc., 2002). Sulfadoxine-pyrimethamine can cause hemolysis in glucose-6-phosphate dehydrogenase-deficient individuals and kernicterus for the baby if given to the mother at term or during nursing period. Severe, but very rarely reported reactions include Stevens-Johnson syndrome and toxic epidermal necrolysis (sulfadoxine-pyrimethamine) and angioedema , anaphylaxis or severe skin reactions (Zithromax®). Due to the rarity and nature of expected adverse effects, however, the potential benefits of participating in the study outweigh the potential harmful effects.

The mechanism of action of azithromycin is different from that of anti-folate antibiotics (sulfadoxine-pyrimethamine, SP). In a phase 2/3 drug interaction study conducted by Pfizer, Inc., administration of trimethoprim/sulfamethoxazole DS (160 mg/800 mg), a similar combination as SP, for 7 days to healthy subjects with co-administration of 1,200 mg azithromycin on the 7th day had no significant effects on peak concentrations or total

exposure or urinary excretion of either trimethoprim or sulfamethoxazole. Serum concentrations of azithromycin following administration of a single 1,200 mg dose after administration of trimethoprim/sulfamethoxazole DS for 7 days were similar to those produced following a 1,200 mg dose of azithromycin in other studies (Pfizer Inc., 2002). Therefore, synergistic toxicity or drug interaction are not likely to follow co-administration of azithromycin and sulfadoxine-pyrimethamine.

In order to avoid any teratogenic effects and kernicterus of the newborn, no study drugs are given before 14 or after 37 completed gestation weeks.

Any adverse reactions arising during the study are treated within the national health system, i.e. there is no special health insurance for the study participants. If neeed, the study team will, however, provide assistance in transportation to appropriate national health care facilities.

All adverse effects are documented according to standard operating procedures. Serious adverse effects are immediately reported to the principal investigators, who will notify the external reviewers about them. Individuals experiencing such a reaction are withdrawn from the study and the external reviewers and principal investigators will jointly decide about the continuation of enrolment and follow-up for others.

5.3 Review board

Four external reviewers (Dr. Michael Boele van Hensbroek and Dr. Sarah A White, Malawi-Liverpool-Wellcome Trust Clinical Research Programme, Blantyre, Malawi, Dr. Yolande Coombes, Blantyre Integrated Malaria Initiative, Malawi, and Prof. Elizabeth Molyneux, Department of Paediatrics, College of Medicine, Malawi) will oversee the progress of the study and assess the safety of the intervention (in interim analysis and in case of any serious adverse effects). All reviewers have extensive experience on malaria research in Malawi. The review board meetings may also be attended as observers by a representative of the research team and a representative of the pharmaceutical company manufacturing and donating azithromycin (Pfizer Inc).

5.4 Study discontinuation criteria

5.4.1 Discontinuation of individual subjects

The participant mother can decide to discontinue the study at any point without giving a reason for her decision and without this having any negative impact of her continued medical care. To control for a potential bias due to losses of follow-up, information on the duration of pregnancy at birth, birth weight and infant's size at six months of age shall be sought (with permission) also from those participants, who otherwise discontinue follow-up.

Individuals experiencing serious adverse effects, becoming seriously ill, meeting the criteria for infant malnutrition (WAZ<-2), or severely violating the protocol are withdrawn from the study by the investigator (but included in the analysis). In such occasions, the reason for

withdrawal will be given and specified in the Case Report Forms. Information on the duration of pregnancy at birth, birth weight and infant's size at six months of age shall be sought (with permission) also from these participants.

5.4.2 Discontinuation of the whole study

The principal investigator and/or the review board have the right to terminate the study if the incidence and/or severity of adverse events overweigh the benefits of the study.

5.5 Potential public health consequenses of the study

5.5.1 Cost of the intervention

Azithromycin is rather expensive, which limits its current national use. The manufacturer of the drug, Pfizer Inc, has, however, previous experience from special accessibility programmes in low-income countries for selected indications (trachoma). Similar arrangements may possibly be negotiated if the current intervention proves successful. This, together with international activities, such as the development of the Global Fund, are believed to make the drug accessible to Malawians, if it proves medically indicated.

If the new antenatal intervention is beneficial over the standard antenatal care (as evidenced by health benefits without adverse effects to the mother or infant), and the regional health authorities wish to change the antimicrobial policy accordingly, the local population will be guaranteed with free access to the same intervention for three years. To ensure this, the manufacturer of azithromycin (Pfizer Inc, Ltd) will provide Lungwena Health Centre with 7500 doses (1 g each) of free Zithromax® (1000 – 1500 pregnancies / year, 2 doses / pregnancy, 3 years). Free sulfadoxine-pyrimethamine will continue to come from the Malawian Ministry of Health and Population, but the study team is committed to assist it in temporary provision problems during the three years after the trial.

5.5.2 Potential induction of antimicrobial resistance

One further public health problem needs to be addressed if the intervention with azithromycin proves efficient on an individual level. This is the potential for development of macrolide (especially erythromycin) -resistant *S. pneumoniae* strains in azithromycin-treated women and the spread of these strains to children, whose respiratory infections are often treated with erythromycin in Malawi. Although azithromycin-therapy in children has been associated with the detection of antimicrobial resistance in treated individuals (Leach et al., 1997), changes in microbial flora are minimal and antibiotic pressure does not seem to significantly increase the population-level prevalence of resistant strains, even in conditions where azithromycin is widely used for a population-based control of endemic trachoma (Fry et al, 2002; Matute et al., 2002; Knirsh C, personal communication). Furthermore, the spread of -antibiotic-resistant bacterial strains from mothers to their children is unlikely unless the therapy is given to children themselves. Finally, the respiratory carriage rate of *S. pneumoniae* among adults in Malawi is only 10% (M. Molyneux, personal communication), further decreasing the possibility of mother-to-child transmission of macrolide resistant bacteria, even if all pregnant women received presumptive STI treatment with azithromycin.

6. Possible constraints

The possible problems with enrolment include hesitation over blood specimens and HIV-testing. Based on our earlier findings, however, neither is likely to produce a significant threat to enrolment, especially since the enrolment rate is already estimated to be much lower than in earlier studies from the same area. Compliance with study protocol may be affected by the high number of individual drug tablets the participants have to take at some ANC visits (maximum 5 tablets), because of which they are taken under direct observation.

Heavy rains between January and March may prevent the participants from attending the antenatal clinic on exactly the agreed dates, but the delays are expected to be non-significant and the research team can provide transportation in critical situations. In case of a serious famine, migration to Mozambique will become more common and loss to follow-up can double from the expected 10%.

Nevirapine tablets are given to HIV-infected individuals during pregnancy by the study nurse offering the post-test counseling. The women are advised to take the tablet during delivery. To avoid stigmatization by HIV status, those women testing negative will be given a placebo tablet (containing Vitamin C). All deliveries are noted by traditional birth attendants (TBA) to the study office within 48 hours, after which the study nurse allocates and sends (with TBAs) nevirapine suspension to the newborns of HIV-infected and identical looking placebo-suspension to the babies of HIV-negative women.

7. Training provided

The project involves two post-graduate students, one from Malawi and one from Finland, who will work for a PhD degree at the university of Tampere (Finland). Both of these students have a 2-year personal stipend covered from the proposed budget and additional funding will be sought during the study (estimated total duration of PhD studies is 4 years). The Malawian PhD student can analyse the economic feasibility of the planned intervention, which would strenghten the health economics knwoledgebase at the COM Department of Community Health.

Through the study, all researchers and research assistants will gain experience in running a randomised trial according to Good Clinical Practice. The health centre nurses will learn to do gestational ultrasounds and the laboratory assistant will practice measuring haemoglobin levels of pregnant women.

Within the framework of the study opportunities exist for COM medical student or MPH projects on KAP to malaria or STI; evaluation of uptake and use of bed nets in the area, contact tracing for patients with STI etc. Such projects would be the subject of separate COMREC applications.

8. Budget and its justification

Budget removed from this version of the protocol

9. References

Anderson SL, Berman J, Kuschner R, Wesche D, Magill A, Wellde B, Schneider I, Dunne M, Schuster BG. Prophylaxis of Plasmodium falciparum malaria with azithromycin administered to volunteers. Ann Intern Med 1995;123:771-3

Bloland P, Slutsker L, Steketee RW, Wirima JJ, Heymann DL, Breman JG. Rates and risk factors for mortality during the first two years of life in rural Malawi. Am J Trop Med Hyg 1996;55:82-6

Centers for Disease Control and Prevention. Sexually transmitted diseases treatment guidelines 2002. MMWR 2002;51(RR-6):1-82

Fry AM, Jha HC, Lietman TM, Chaudhary JS, Bhatta RC, Elliott J, Hyde T, Schuchat A, Gaynor B, Dowell SF. Adverse and beneficial secondary effects of mass treatment with azithromycin to eliminate blindness due to trachoma in Nepal. Clin Infect Dis 2002;25:395-402

Government of Malawi. Malaria Policy. National Malaria Control Programme, Community Health Sciences Unit, Division of Preventive Health Services, Ministry of Health and Population. 2002

Gray RH, Wabwire-Mangen F, Kigozi G, Sewankambo NK, Serwadda D, Moulton LH, Quinn TC, O'Brien KL, Meehan M, Abramowsky C, Robb M, Wawer MJ. Randomized trial of presumptive sexually transmitted disease therapy during pregnancy in Rakai, Uganda. Am J Obstet Gynecol 2001;185:1209-17

Gruber F, Brajac I, Jonjic A, Grubisic-Greblo H, Lenkovic M, Stasic A. Comparative trial of azithromycin and ciprofloxacin in the treatment of gonorrhea. J Chemother 1997;9:263-6

Hauth JC, Goldenberg RL, Andrews WW, DuBard MB, Copper RL. Reduced incidence of preterm delivery with metronidatzole and erythromycin in women with bacterial vaginosis. N Engl J Med 1995;333:1732-6

Kulmala T, Vaahtera M, Ndekha M, Koivisto A-M, Cullinan T, Salin M-L, Ashorn P. The importance of preterm births for peri- and neonatal mortality in rural Malawi. Pediatr Perinat Epid 2000;14:219-26

Kulmala T, Vaahtera M, Ndekha M, Cullinan T, Salin M-L Koivisto A-M, Ashorn P. Gestational health and predictors of newborn weight amongst pregnant women in rural Malawi. Afr J Reprod Health 2001;5:99-108

Leach AJ, Shelby-James TM, Mayo M, Gratten M, Laming AC, Currie BJ, Mathews JD. A prospective study of the impact of community-based azithromycin treatment of trachoma on carriage and resistance of Streptococcus pneumoniae. Clin Infect Dis 1997;24:356-62

Lamont RF. The prevention of preterm birth with the use of antibiotics. Eur J Pediatr 1999;158:S2-4

Matute AJ, Schurink CA, Krijnen RM, Florijn A, Rozenberg-Arska M, Hoepelman IM. Double-blind, placebo-controlled study comparing the effect of azithromycin with clarithromycin on oropharyngeal and bowel microflora in volunteers. Eur J Clin Microbiol Infect Dis 2002;21:427-31.

Meuris S, Piko BB, Eerens P, Vanbellinghen AM, Dramaix M, Hennart P. Gestational malaria: assessment of its consequences on fetal growth. Am J Trop Med Hyg 1993;48:603-9

National AIDS Control Commission Malawi. Sentinel surveillance report 2001. HIV / syphilis seroprevalence in antenatal clinic attendees. National AIDS Control Commission, Lilongwe, 2001

National Statistical Office (Malawi) and ORC Macro. Malawi Demographic and Health Survey 2000. Zomba, Malawi and Calverton, Maryland, USA: National Statistical Office and ORC Macro; 2001

Nzila AM, Mberu EK, Sulo J, Dayo H, Winstanley PA, Sibley CH, Watkins WM. Towards an understanding of the mechanism of pyrimethamine-sulfadoxine resistance in Plasmodium falciparum: genotyping of dihydrofolate reductase and dihydropteroate synthase of Kenyan parasites. Antimicrob Agents Chemoter 2000;44:991-6

Odugbemi T, Oyewole F, Isichei CS, Onwukeme KE, Adeyemi-Doro FA. Single oral dose of azithromycin for therapy of susceptible sexually transmitted diseases: a multicenter open evaluation. West Afr J Med 1993;12:136-40

Ohrt C, Willingmyre GD, Lee P, Knirsch C, Milhous W. Assessment of azithromycin in combination with other antimalarial drugs against Plasmodium falciparum in vitro. Antimicrob Agents Chemother 2002:46:2518-24

Osman NB, Challis K, Cotiro M, Nordahl G, Bergström S. Perinatal outcome in an obstetric cohort of Mozambican women. J Trop Pediatr 2001;47:30-8

Parise ME, Ayisi JG, Nahlen BL, Schultz LJ, Roberts JM, Misore A, Muga R, Oloo AJ, Steketee RW. Efficacy of sulfadoxine-pyrimethamine for prevention of placental malaria in an area of Kenya with a high prevalence of malaria and human immunodeficiency virus infection. Am J Trop Med Hyg 1998;59:813-22

Pfizer Inc. Zithromax® product information [cited 2002 Aug 21] Available from: URL: http://www.pfizer.com/hml/pi's/zithromaxoralpi.pdf

Pfizer Pharmaceuticals Group. International Product Information for azithromycin. Pfizer Inc., Mew York, 2002

Regan JA, Klebanoff MA, Nugent RB, Eschenbach DA, Blackwelder WC, Lou Y, Gibbs RS, Rettig PJ, Martin DH, Edelman R. Colonization with group B streptococci in pregnancy and adverse outcome. VIP Study Group. Am J Obstet Gynecol 1996;174:1354-60

Roche Pharmaceuticals. Fansidar® Complete product information. [cited 2002 Aug 21]

Available from: URL: http://www.rocheusa.com/products/fansidar/pi.html

Rogerson SJ, Chaluluka E, Kanjala M, Mkundika P, Mhango C, Molyneux ME. Intermittent sulfadoxine-pyrimethamine in pregnancy: effectiveness against malaria morbidity in Blantyre, Malawi, in 1997-99. Trans R Soc Trop Med Hyg 2000(a);94:549-53

Rogerson SJ, van den Broek NR, Chaluluka E, Qongwane C, Mhango CG, Molyneux ME. Malaria and anemia in antenatal women in Blantyre, Malawi: a twelve-month survey. Am J Trop Med 2000(b);62:335-40

Schultz LJ, Steketee RW, Macheso A, Kazembe P, Chitsulo L, Wirima JJ. The efficacy of antimalarial regimens containing sulfadoxine-pyrimethamine and/or chloroquine in preventing peripheral and placental Plasmodium falciparum infection among pregnant women in Malawi. Am J Trop Med Hyg 1994;51:515-22

Shulman CE, Dorman EK, Cutts F, Kawuondo K, Bulmer JN, Peshu N, Marsh K. Intermittent sulphadoxine-pyrimethamine to prevent severe anaemia secondary to malaria in pregnancy: a randomised placebo-controlled trial. Lancet 1999;353:632-6

Shulman CE, Marshall T, Dorman EK, Bulmer JN, Cutts F, Peshu N, Marsh K. Malaria in pregnancy: adverse effects on haemoglobin levels and birthweight in primigravidae and multigravidae. Trop Med Int Health 2001;6:770-8

Sibley CH, Hyde JE, Sims PF, Plowe CV, Kublin JG, Mberu EK, Cowman AF, Winstanley PA, Watkins WM, Nzila AM. Pyrimethamine-sulfadoxine resistence in Plasmodium falciparum: what next ? Trends Parasitol 2001;17:582-8

Steketee RW, Wirima JJ, Slutsker L, Breman JG, Heymann DL. Comparability of treatment groups and risk factors for parasitemia at the first antenatal clinic visit in a study of malaria treatment and prevention in pregnancy in rural Malawi. Am J Trop Med 1996;55:17-23

Sullivan AD, Nyirenda T, Cullinan T, Taylor T, Harlow SD, James SA, Meshnick SR. Malaria infection during pregnancy: intrauterine retardation and preterm delivery in Malawi. J Infect Dis 1999 ;179:1580-3

Swanston WH, Prabhakar P, Barrow L, Mahabir BS, Furlonge C. Single dose (direct observed) azithromycin therapy for Neisseria gonorrhoeae and Chlamydia trachomatis in STD clinic attenders with genital discharge in Trinidad and Tobago. West Indian Med J 2001;50:198-202

Taylor WR, Richie TL, Fryauff DJ, Picarima H, Ohrt C, Tang D, Braitman D, Murphy GS, Widjaja H, Tjitra E, Ganjar A, Jones TR, Basri H, Berman J. Malaria prophylaxis using azithromycin: a double-blind, placebo-controlled trial in Irian Jaya, Indonesia. Clin Infect Dis 1999;28:74-81

Temmerman M, Njagi E, Nagelkerke N, Ndinya-Achola J, Plummer FA, Meheus A. Mass antimicrobial treatment in pregnancy. A randomized, placebo-controlled trial in a population with high rates of sexually transmitted diseases. J Reprod Med 1995;40:176-80

Tsui A, Graft-Johnson D, Bisika T. Assessing perceived risks for pregnancy and sexually transmitted infection among men and women in Malawi. [cited 2002 Aug 26] Available from: URL: http://www.cpc.unc.edu/measure/research/studies/assessing.html

United Nations Children's Fund (UNICEF): The state of the world's children 2001. New York: UNICEF; 2001

Verhoeff FH, Milligan P, Brabin BJ, Mlanga S, Nakoma V. Gestational age assessment by nurses in a developing country using the Ballard method, external criteria only. Ann Trop Paediatr 1997;17:333-42

Verhoeff FH, Brabin BJ, Chimsuku L, Kazembe P, Russell WB, Broadhead RL. An evaluation of the effects of intermittent sulfadoxine-pyrimethamine treatment in pregnancy on parasite clearance and risk of low birthweight in rural Malawi. Ann Trop Med Parasitol 1998;92:141-50

Verhoeff FH, Brabin BJ, Chimsuku L, Kazembe P, Broadhead RL. An analysis of the determinants of anaemia in pregnant women in rural Malawi – a basis for action. Ann Trop Med Parasitol 1999;93:119-33

Waugh MA. Open study of the safety and efficacy of a single oral dose of azithromycin for the treatment of uncomplicated gonorrhoea in men and women. J Antimicrob Chemother 1993;31 (Suppl E):193-8

Wolfe EB, Parise ME, Haddix AC, Nahlen BL, Ayisi JG, Misore A, Steketee RW. Cost-effectiveness of sulfadoxine-pyrimethamine for the prevention of malaria-associated low birth weight. Am J Trop Med Hyg 2001;64:178-86

10. Appendixes

Appendix 01. Participant information form
Appendix 02. Identification and informed consent form
Appendix 03. Socio-economic background form
Appendix 04. Mother's health history form
Appendix 05. First visit antenatal examination form
Appendix 06. Ultrasound examination form
Appendix 07. Laboratory measurements form
Appendix 08. Inclusion criteria and randomisation form
Appendix 09. Treatments form
Appendix 10. Follow-up antenatal examination form
Appendix 11. Delivery information-mother form
Appendix 12. Delivery information-attendant form
Appendix 13. Delivery information-newborn form
Appendix 14. Maternal post-delivery follow-up form
Appendix 15. Child follow-up form
Appendix 16. Adverse event report form form
Appendix 17. Non-scheduled visit information form
