# Supplementary material for: The impact of maternal antenatal treatment with two doses of azithromycin and monthly sulphadoxine-pyrimethamine on child weight, mid-upper arm circumference and head circumference: A randomized controlled trial
Source: PLoS One. 2019 May 7;14(5):e0216536. doi: 10.1371/journal.pone.0216536 (PMC6504037; doi:10.1371/journal.pone.0216536)
Supplement: S1 Table — (DOCX) [file pone.0216536.s003.docx]

**Table S1. Baseline characteristics of all women enrolled to the study at enrollment, by study group**

| **Characteristic** | **Control (SP twice) (N=436),**  **n (%)** | **Monthly SP, (N=441),**  **n (%)** | **AZI-SP (N=443),**  **n (%)** |
| --- | --- | --- | --- |
| Age, years, mean (SD) | 25 (7) | 25 (7) | 25 (6) |
| Height, cm, mean (SD) | 155.0 (5.5) | 154.8 (5.4) | 155.3 (5.6)^a^ |
| BMI, kg/m^2^, mean (SD) | 21.7 (2.2) | 21.8 (2.1) | 21.9 (2.1)^a^ |
| Gestational age at enrollment, weeks, mean (SD) | 20.3 (3.0) | 20.0 (3.2) | 20.0 (3.0) |
| Primiparous | 110 (25.2%) | 107 (24.3%) | 89 (20.1%) |
| HIV positive | 48/396 (12.1%) | 64/400 (16.0%) | 49/398 (12.3%) |
| Positive syphilis test result Treponema pallidum hemagglutination assay | 18/433 (4.2%) | 27/435 (6.2%) | 21/440 (4.8%) |
| Blood Hb concentration, g/L, mean (SD) | 110 (19) | 111 (17) | 110 (20) |
| Moderate or severe anemia, Hb < 100 g/L | 116 (26.6%) | 106 (24.0%) | 129 (29.1%) |
| Severe anemia, Hb < 70 g/L | 9 (2.1%) | 2 (0.5%) | 9 (2.0%) |
| Microscopic peripheral blood malaria parasitemia | 49/435 (11.3%) | 41 (9.3%) | 27 (6.1%) |
| Literate mothers | 116 (26.6%) | 129 (29.3%) | 139 (31.4%) |
| Years of schooling completed, median (interquartile range) | 0 (0, 4)^a^ | 0 (0, 4) | 2 (0, 4) |
| Proxy for socio-economic status^b^ | -0.05 (0.90) | 0.03 (1.08) | 0.02 (1.01) |

SP = sulfadoxine-pyrimethamine. AZI-SP = intervention group with monthly SP and two doses of azithromycin. BMI = body-mass index. HIV = human immunodeficiency virus. Hb = hemoglobin.

^a^ Value missing for one participant

^b^ Created with principal component analysis by combining information on the building material of the house, main source of water, sanitary facility and ownership of household items.
