# Supplementary material for: The impact of maternal antenatal treatment with two doses of azithromycin and monthly sulphadoxine-pyrimethamine on child weight, mid-upper arm circumference and head circumference: A randomized controlled trial
Source: PLoS One. 2019 May 7;14(5):e0216536. doi: 10.1371/journal.pone.0216536 (PMC6504037; doi:10.1371/journal.pone.0216536)
Supplement: S2 Table — (DOCX) [file pone.0216536.s004.docx]

**Table S2. Baseline characteristics of participating women at enrolment and infant characteristics at birth, by follow-up status at 60 months**

| **Characteristic** | **Anthropometric measurement done at 60mo (N=953), n (%)** | **Lost to follow-up at 60mo (N=316), n (%)** | **P-value^a^** |
| --- | --- | --- | --- |
| Age, years, mean (SD) | 25 (6) | 24 (6) | 0.100 |
| Height, cm, mean (SD) | 155.1 (5.5)  (1 missing data) | 155.0 (5.7) | 0.764 |
| BMI, kg/m^2^, mean (SD) | 21.8 (2.2)  (1 missing data) | 21.7 (2.2) | 0.668 |
| Gestational age at enrollment, weeks, mean (SD) | 20.2 (3.1) | 19.8 (3.1) | 0.039 |
| Primiparous | 215 (22.6%) | 78 (24.7%) | 0.442 |
| HIV positive | 86/864 (10.0%) | 68/218 (24.2%) | <0.001 |
| Positive syphilis test result Treponema pallidum hemagglutination assay | 45/945 (4.8%) | 17/313 (5.4%) | 0.652 |
| Blood Hb concentration, g/L, mean (SD) | 111 (18) | 109 (19) | 0.149 |
| Moderate or severe anemia, Hb < 100 g/L | 243 (25.5%) | 96 (30.4%) | 0.092 |
| Severe anemia, Hb < 70 g/L | 10 (1.1%) | 7 (2.2%) | 0.154 |
| Microscopic peripheral blood malaria parasitemia | 74 (7.8%) | 35/315 (11.1%) | 0.081 |
| Literate mothers | 268 (28.1%) | 101 (32.0%) | 0.199 |
| Years of schooling completed, median (interquartile range) | 1 (0, 4) | 1 (0, 4)  (1 missing data) | 0.350 |
| Proxy for socio-economic status^b^ | 0.00 (1.00) | -0.01 (1.01) | 0.924 |
| **Infant characteristics** |  |  |  |
| Duration of pregnancy, mean (SD) | 38.8 (1.7) | 38.4 (2.3)  (4 missing data) | 0.004 |
| Birth weight, kg, mean (SD) | 2.98 (0.47)  (31 missing data) | 2.88 (0.47)  (39 missing data) | 0.001 |
| Low birth weight, <2.5 kg | 82/922 (8.9%) | 38/277 (13.7%) | 0.022 |
| Small for gestational age^c^ | 165/919 (18.0%) | 58/276 (21.0%) | 0.253 |

BMI = body-mass index. HIV = human immunodeficiency virus. Hb = hemoglobin.

^a^ For continuous variable P-value derived from regression analysis, and for dichotomous outcomes from Fisher’s exact test

^b^ Created with principal component analysis by combining information on the building material of the house, main source of water, sanitary facility and ownership of household items.

^c^ Small for gestational age calculated using INTERGROWTH-21^st^ Project standards for newborn size by gestational age, defined as birthweight-for-gestation-week < 10^th^ centile.
