# Supplementary material for: The impact of maternal antenatal treatment with two doses of azithromycin and monthly sulphadoxine-pyrimethamine on child weight, mid-upper arm circumference and head circumference: A randomized controlled trial
Source: PLoS One. 2019 May 7;14(5):e0216536. doi: 10.1371/journal.pone.0216536 (PMC6504037; doi:10.1371/journal.pone.0216536)
Supplement: S3 Table — (DOCX) [file pone.0216536.s005.docx]

**S3 Table. Mean (SD) weight, weight-for-age Z-score (WAZ), and weight-for-height Z-score (WHZ) by intervention group at one, six, 12, 24, 36, 48, and 60 months of age.**

| **Outcome** | **Age** | **Mean (SD)** | | | | **Comparison between AZI-SP and control group** | | **Comparison between AZI-SP and monthly SP group** | | **Comparison between monthly SP and control group** | |
| --- | --- | --- | --- | --- | --- | --- | --- | --- | --- | --- | --- |
|  |  | **Control** | **Monthly SP** | **AZI-SP** | **Overall p-value** | **Difference in means  (95% CI)** | **P-value** | **Difference in means  (95% CI)** | **P-value** | **Difference in means  (95% CI)** | **P-value** |
| Mean (SD) weight (kg) | 1 mo | 3.96 (0.60) | 4.03 (0.62) | 4.08 (0.52) | 0.014 | 0.12 (0.04 to 0.20) | 0.003 | 0.05 (-0.03 to 0.13) | 0.253 | 0.07 (-0.01 to 0.16) | 0.104 |
|  | 1 mo, adjusted^a^ | - | - | - | 0.086 | 0.08 (0.01 to 0.16) | 0.034 | 0.01 (-0.07 to 0.09) | 0.749 | 0.07 (-0.01 to 0.15) | 0.102 |
|  | 1 mo, imputed^b^ | 3.96 (0.60) | 4.02 (0.62) | 4.07 (0.52) | 0.016 | 0.11 (0.04 to 0.19) | 0.004 | 0.04 (-0.03 to 0.12) | 0.271 | 0.07 (-0.01 to 0.15) | 0.105 |
|  | 6 mo | 7.05 (0.94) | 7.12 (1.02) | 7.24 (0.97) | 0.024 | 0.19 (0.05 to 0.33) | 0.007 | 0.12 (-0.02 to 0.27) | 0.093 | 0.07 (-0.08 to 0.21) | 0.355 |
|  | 6 mo, adjusted^a^ | - | - | - | 0.074 | 0.16 (0.02 to 0.29) | 0.023 | 0.07 (-0.07 to 0.22) | 0.310 | 0.08 (-0.06 to 0.22) | 0.258 |
|  | 6 mo, imputed^b^ | 7.05 (0.93) | 7.09 (1.01) | 7.22 (0.97) | 0.032 | 0.17 (0.04 to 0.31) | 0.012 | 0.13 (-0.01 to 0.27) | 0.064 | 0.04 (-0.10 to 0.18) | 0.550 |
|  | 12 mo | 8.36 (1.08) | 8.46 (1.11) | 8.50 (1.08) | 0.198 | 0.14 (-0.02 to 0.30) | 0.077 | 0.04 (-0.12 to 0.21) | 0.587 | 0.10 (-0.07 to 0.26) | 0.237 |
|  | 12 mo, adjusted^a^ | - | - | - | 0.096 | 0.15 (-0.01 to 0.31) | 0.059 | 0.00 (-0.16 to 0.16) | 0.999 | 0.15 (-0.01 to 0.32) | 0.067 |
|  | 12 mo, imputed^b^ | 8.35 (1.06) | 8.44 (1.09) | 8.48 (1.08) | 0.230 | 0.13 (-0.02 to 0.28) | 0.091 | 0.05 (-0.11 to 0.20) | 0.555 | 0.08 (-0.07 to 0.24) | 0.277 |
|  | 24 mo | 10.17 (1.16) | 10.33 (1.33) | 10.30 (1.24) | 0.197 | 0.13 (-0.05 to 0.31) | 0.148 | -0.02 (-0.22 to 0.17) | 0.815 | 0.16 (-0.03 to 0.35) | 0.105 |
|  | 24 mo, adjusted^a^ | - | - | - | 0.286 | 0.11 (-0.07 to 0.29) | 0.232 | -0.03 (-0.21 to 0.16) | 0.758 | 0.14 (-0.05 to 0.32) | 0.139 |
|  | 24 mo, imputed^b^ | 10.17 (1.14) | 10.31 (1.32) | 10.33 (1.23) | 0.134 | 0.16 (-0.01 to 0.34) | 0.062 | 0.03 (-0.16 to 0.21) | 0.777 | 0.14 (-0.04 to 0.32) | 0.133 |
|  | 36 mo | 12.03 (1.40) | 12.12 (1.49) | 12.13 (1.45) | 0.595 | 0.10 (-0.12 to 0.32) | 0.355 | 0.01 (-0.21 to 0.23) | 0.947 | 0.10 (-0.13 to 0.32) | 0.404 |
|  | 36 mo, adjusted^a^ | - | - | - | 0.683 | 0.10 (-0.12 to 0.32) | 0.384 | 0.04 (-0.18 to 0.27) | 0.709 | 0.06 (-0.17 to 0.28) | 0.625 |
|  | 36 mo, imputed^b^ | 12.02 (1.37) | 12.10 (1.47) | 12.11 (1.47) | 0.612 | 0.10 (-0.11 to 0.31) | 0.354 | 0.02 (-0.20 to 0.23) | 0.879 | 0.08 (-0.13 to 0.29) | 0.444 |
|  | 36 mo, imputed^c^ | 12.02 (1.39) | 12.12 (1.49) | 12.13 (1.44) | 0.608 | 0.10 (-0.12 to 0.32) | 0.358 | 0.01 (-0.21 to 0.23) | 0.919 | 0.09 (-0.13 to 0.31) | 0.428 |
|  | 48 mo | 13.77 (1.56) | 13.77 (1.65) | 13.85 (1.72) | 0.808 | 0.07 (-0.18 to 0.33) | 0.565 | 0.07 (-0.18 to 0.33) | 0.572 | 0.00 (-0.25 to 0.25) | 1.000 |
|  | 48 mo, adjusted^a^ | - | - | - | 0.649 | 0.08 (-0.18 to 0.33) | 0.544 | 0.12 (-0.14 to 0.38) | 0.357 | -0.04 (-0.29 to 0.20) | 0.736 |
|  | 48 mo, imputed^b^ | 13.78 (1.54) | 13.76 (1.68) | 13.82 (1.71) | 0.904 | 0.04 (-0.20 to 0.28) | 0.749 | 0.06 (-0.19 to 0.30) | 0.661 | -0.02 (-0.26 to 0.23) | 0.899 |
|  | 48 mo, imputed^c^ | 13.77 (1.55) | 13.78 (1.63) | 13.85 (1.71) | 0.829 | 0.07 (-0.18 to 0.32) | 0.584 | 0.07 (-0.19 to 0.32) | 0.601 | 0.00 (-0.25 to 0.25) | 0.986 |
|  | 60 mo | 15.25 (1.61) | 15.32 (1.72) | 15.49 (1.86) | 0.236 | 0.23 (-0.04 to 0.50) | 0.094 | 0.17 (-0.11 to 0.45) | 0.246 | 0.07 (-0.20 to 0.33) | 0.621 |
|  | 60 mo, adjusted^a^ | - | - | - | 0.277 | 0.22 (-0.05 to 0.50) | 0.109 | 0.13 (-0.15 to 0.41) | 0.357 | 0.09 (-0.17 to 0.36) | 0.488 |
|  | 60 mo, imputed^b^ | 15.27 (1.62) | 15.32 (1.71) | 15.45 (1.86) | 0.411 | 0.17 (-0.09 to 0.44) | 0.194 | 0.13 (-0.14 to 0.39) | 0.346 | 0.05 (-0.21 to 0.30) | 0.713 |
|  | 60 mo, imputed^c^ | 15.25 (1.60) | 15.32 (1.71) | 15.48 (1.85) | 0.234 | 0.23 (-0.04 to 0.50) | 0.093 | 0.16 (-0.12 to 0.44) | 0.249 | 0.07 (-0.20 to 0.33) | 0.611 |
| Mean (SD) WAZ | 1 mo | -0.87 (1.06) | -0.78 (1.08) | -0.59 (0.96) | <0.001 | 0.28 (0.14 to 0.43) | <0.001 | 0.19 (0.04 to 0.33) | 0.011 | 0.10 (-0.05 to 0.25) | 0.212 |
|  | 1 mo, adjusted^a^ | - | - | - | 0.015 | 0.20 (0.07 to 0.34) | 0.004 | 0.09 (-0.06 to 0.23) | 0.236 | 0.12 (-0.03 to 0.27) | 0.121 |
|  | 1 mo, imputed^b^ | -0.87 (1.06) | -0.77 (1.08) | -0.60 (0.96) | <0.001 | 0.28 (0.14 to 0.42) | <0.001 | 0.18 (0.04 to 0.32) | 0.014 | 0.10 (-0.05 to 0.25) | 0.192 |
|  | 6 mo | -0.79 (1.13) | -0.74 (1.21) | -0.55 (1.15) | 0.010 | 0.24 (0.08 to 0.41) | 0.004 | 0.20 (0.03 to 0.37) | 0.025 | 0.05 (-0.12 to 0.22) | 0.597 |
|  | 6 mo, adjusted^a^ | - | - | - | 0.117 | 0.17 (0.01 to 0.33) | 0.038 | 0.09 (-0.09 to 0.26) | 0.332 | 0.09 (-0.09 to 0.26) | 0.338 |
|  | 6 mo, imputed^b^ | -0.78 (1.12) | -0.77 (1.21) | -0.57 (1.15) | 0.015 | 0.21 (0.05 to 0.37) | 0.009 | 0.20 (0.03 to 0.36) | 0.018 | 0.01 (-0.15 to 0.18) | 0.861 |
|  | 12 mo | -1.03 (1.12) | -0.95 (1.12) | -0.86 (1.14) | 0.154 | 0.16 (0.00 to 0.33) | 0.053 | 0.08 (-0.08 to 0.25) | 0.328 | 0.08 (-0.09 to 0.25) | 0.342 |
|  | 12 mo, adjusted^a^ | - | - | - | 0.110 | 0.16 (-0.01 to 0.32) | 0.066 | 0.00 (-0.17 to 0.17) | >0.999 | 0.16 (-0.02 to 0.33) | 0.075 |
|  | 12 mo, imputed^b^ | -1.02 (1.09) | -0.963 (1.10) | -0.88 (1.14) | 0.186 | 0.15 (-0.01 to 0.31) | 0.066 | 0.08 (-0.08 to 0.24) | 0.311 | 0.07 (-0.09 to 0.22) | 0.406 |
|  | 24 mo | -1.36 (0.96) | -1.27 (1.07) | -1.24 (1.03) | 0.295 | 0.11 (-0.04 to 0.26) | 0.136 | 0.03 (-0.13 to 0.19) | 0.727 | 0.09 (-0.07 to 0.24) | 0.272 |
|  | 24 mo, adjusted^a^ | - | - | - | 0.365 | 0.08 (-0.07 to 0.24) | 0.275 | -0.02 (-0.18 to 0.14) | 0.808 | 0.10 (-0.05 to 0.26) | 0.188 |
|  | 24 mo, imputed^b^ | -1.35 (0.94) | -1.28 (1.05) | -1.21 (1.03) | 0.157 | 0.14 (0.00 to 0.28) | 0.057 | 0.06 (-0.09 to 0.21) | 0.425 | 0.08 (-0.07 to 0.23) | 0.291 |
|  | 36 mo | -1.36 (0.96) | -1.32 (1.00) | -1.29 (0.99) | 0.614 | 0.08 (-0.07 to 0.22) | 0.325 | 0.03 (-0.12 to 0.18) | 0.676 | 0.04 (-0.11 to 0.20) | 0.577 |
|  | 36 mo, adjusted^a^ | - | - | - | 0.722 | 0.06 (-0.09 to 0.22) | 0.420 | 0.03 (-0.12 to 0.19) | 0.680 | 0.03 (-0.12 to 0.19) | 0.696 |
|  | 36 mo, imputed^b^ | -1.37 (0.94) | -1.33 (0.98) | -1.30 (1.01) | 0.646 | 0.07 (-0.08 to 0.21) | 0.351 | 0.03 (-0.12 to 0.18) | 0.703 | 0.04 (-0.10 to 0.18) | 0.580 |
|  | 36 mo, imputed^c^ | -1.36 (0.95) | -1.32 (1.00) | -1.29 (0.99) | 0.618 | 0.07 (-0.07 to 0.22) | 0.327 | 0.03 (-0.11 to 0.18) | 0.646 | 0.04 (-0.11 to 0.19) | 0.609 |
|  | 48 mo | -1.31 (0.89) | -1.33 (0.93) | -1.28 (0.98) | 0.816 | 0.03 (-0.11 to 0.18) | 0.649 | 0.05 (-0.10 to 0.19) | 0.535 | -0.01 (-0.15 to 0.13) | 0.860 |
|  | 48 mo, adjusted^a^ | - | - | - | 0.747 | 0.03 (-0.12 to 0.18) | 0.703 | 0.06 (-0.09 to 0.21) | 0.445 | -0.03 (-0.17 to 0.11) | 0.688 |
|  | 48 mo, imputed^b^ | -1.30 (0.88) | -1.33 (0.94) | -1.29 (0.97) | 0.858 | 0.02 (-0.12 to 0.16) | 0.802 | 0.04 (-0.10 to 0.18) | 0.579 | -0.02 (-0.16 to 0.11) | 0.754 |
|  | 48 mo, imputed^c^ | -1.31 (0.88) | -1.32 (0.91) | -1.28 (0.97) | 0.840 | 0.03 (-0.11 to 0.18) | 0.669 | 0.04 (-0.10 to 0.19) | 0.567 | -0.01 (-0.15 to 0.13) | 0.880 |
|  | 60 mo | -1.37 (0.78) | -1.34 (0.82) | -1.27 (0.89) | 0.304 | 0.10 (-0.03 to 0.23) | 0.132 | 0.08 (-0.06 to 0.21) | 0.271 | 0.03 (-0.10 to 0.15) | 0.699 |
|  | 60 mo, adjusted^a^ | - | - | - | 0.387 | 0.09 (-0.04 to 0.23) | 0.169 | 0.05 (-0.09 to 0.18) | 0.497 | 0.05 (-0.08 to 0.17) | 0.475 |
|  | 60 mo, imputed^b^ | -1.35 (0.78) | -1.34 (0.82) | -1.28 (0.89) | 0.510 | 0.07 (-0.05 to 0.20) | 0.264 | 0.06 (-0.07 to 0.18) | 0.383 | 0.02 (-0.11 to 0.14) | 0.801 |
|  | 60 mo, imputed^c^ | -1.37 (0.77) | -1.34 (0.82) | -1.27 (0.88) | 0.298 | 0.10 (-0.03 to 0.23) | 0.128 | 0.08 (-0.06 to 0.21) | 0.272 | 0.03 (-0.10 to 0.15) | 0.685 |
| Mean (SD) WHZ | 1 mo | 0.56 (1.15) | 0.57 (1.15) | 0.61 (1.15) | 0.821 | 0.05 (-0.11 to 0.21) | 0.548 | 0.04 (-0.12 to 0.20) | 0.648 | 0.01 (-0.15 to 0.17) | 0.888 |
|  | 1 mo, adjusted^a^ | - | - | - | 0.982 | 0.02 (-0.16 to 0.19) | 0.848 | 0.01 (-0.17 to 0.19) | 0.907 | 0.01 (-0.17 to 0.18) | 0.942 |
|  | 1 mo, imputed^b^ | 0.56 (1.15) | 0.57 (1.15) | 0.61 (1.16) | 0.790 | 0.05 (-0.11 to 0.21) | 0.513 | 0.04 (-0.12 to 0.20) | 0.615 | 0.01 (-0.15 to 0.17) | 0.878 |
|  | 6 mo | 0.29 (1.18) | 0.31 (1.23) | 0.43 (1.19) | 0.246 | 0.14 (-0.04 to 0.31) | 0.124 | 0.12 (-0.06 to 0.29) | 0.185 | 0.02 (-0.16 to 0.19) | 0.856 |
|  | 6 mo, adjusted^a^ | - | - | - | 0.517 | 0.11 (-0.08 to 0.29) | 0.252 | 0.04 (-0.14 to 0.23) | 0.639 | 0.06 (-0.13 to 0.25) | 0.514 |
|  | 6 mo, imputed^b^ | 0.29 (1.18) | 0.26 (1.25) | 0.41 (1.19) | 0.191 | 0.12 (-0.05 to 0.29) | 0.156 | 0.15 (-0.02 to 0.32) | 0.093 | -0.03 (-0.20 to 0.15) | 0.768 |
|  | 12 mo | -0.16 (1.06) | -0.10 (1.08) | -0.11 (1.08) | 0.711 | 0.05 (-0.11 to 0.21) | 0.540 | -0.01 (-0.17 to 0.14) | 0.856 | 0.06 (-0.10 to 0.22) | 0.431 |
|  | 12 mo, adjusted^a^ | - | - | - | 0.450 | 0.05 (-0.11 to 0.22) | 0.537 | -0.06 (-0.22 to 0.11) | 0.510 | 0.11 (-0.06 to 0.28) | 0.207 |
|  | 12 mo, imputed^b^ | -0.16 (1.06) | -0.12 (1.07) | -0.12 (1.08) | 0.864 | 0.04 (-0.11 to 0.19) | 0.627 | 0.00 (-0.15 to 0.16) | 0.972 | 0.04 (-0.12 to 0.19) | 0.653 |
|  | 24 mo | -0.31 (1.05) | -0.20 (1.09) | -0.27 (1.02) | 0.405 | 0.04 (-0.12 to 0.19) | 0.659 | -0.07 (-0.23 to 0.08) | 0.361 | 0.11 (-0.05 to 0.27) | 0.187 |
|  | 24 mo, adjusted^a^ | - | - | - | 0.249 | 0.01 (-0.15 to 0.17) | 0.928 | -0.12 (-0.28 to 0.04) | 0.153 | 0.12 (-0.04 to 0.29) | 0.144 |
|  | 24 mo, imputed^b^ | -0.27 (1.04) | -0.18 (1.09) | -0.23 (1.02) | 0.489 | 0.05 (-0.11 to 0.20) | 0.546 | -0.05 (-0.20 to 0.11) | 0.533 | 0.10 (-0.06 to 0.25) | 0.229 |
|  | 36 mo | -0.29 (1.12) | -0.25 (1.07) | -0.31 (1.01) | 0.787 | -0.02 (-0.18 to 0.15) | 0.833 | -0.05 (-0.21 to 0.10) | 0.493 | 0.04 (-0.13 to 0.21) | 0.668 |
|  | 36 mo, adjusted^a^ | - | - | - | 0.859 | -0.02 (-0.19 to 0.15) | 0.816 | -0.05 (-0.21 to 0.12) | 0.582 | 0.03 (-0.15 to 0.20) | 0.776 |
|  | 36 mo, imputed^b^ | -0.32 (1.13) | -0.25 (1.07) | -0.31 (1.03) | 0.634 | 0.00 (-0.16 to 0.16) | 0.972 | -0.07 (-0.22 to 0.09) | 0.408 | 0.07 (-0.10 to 0.23) | 0.414 |
|  | 36 mo, imputed^c^ | -0.29 (1.11) | -0.26 (1.07) | -0.31 (1.00) | 0.822 | -0.02 (-0.18 to 0.14) | 0.826 | -0.05 (-0.21 to 0.11) | 0.533 | 0.03 (-0.14 to 0.20) | 0.716 |
|  | 48 mo | -0.27 (1.13) | -0.31 (1.15) | -0.34 (1.10) | 0.707 | -0.07 (-0.24 to 0.10) | 0.410 | -0.02 (-0.20 to 0.15) | 0.778 | -0.05 (-0.22 to 0.13) | 0.599 |
|  | 48 mo, adjusted^a^ | - | - | - | 0.691 | -0.07 (-0.25 to 0.11) | 0.467 | 0.00 (-0.18 to 0.18) | 0.966 | -0.07 (-0.25 to 0.11) | 0.445 |
|  | 48 mo, imputed^b^ | -0.28 (1.13) | -0.34 (1.18) | -0.36 (1.11) | 0.594 | -0.08 (-0.25 to 0.08) | 0.319 | -0.02 (-0.19 to 0.14) | 0.774 | -0.06 (-0.23 to 0.11) | 0.494 |
|  | 48 mo, imputed^c^ | -0.27 (1.12) | -0.31 (1.13) | -0.34 (1.09) | 0.690 | -0.07 (-0.25 to 0.10) | 0.391 | -0.03 (-0.20 to 0.14) | 0.718 | -0.04 (-0.22 to 0.13) | 0.626 |
|  | 60 mo | -0.45 (1.03) | -0.5 (1.03) | -0.43 (1.02) | 0.682 | 0.02 (-0.14 to 0.18) | 0.835 | 0.07 (-0.09 to 0.23) | 0.398 | -0.05 (-0.22 to 0.11) | 0.531 |
|  | 60 mo, adjusted^a^ | - | - | - | 0.853 | 0.03 (-0.14 to 0.19) | 0.743 | 0.05 (-0.12 to 0.21) | 0.576 | -0.02 (-0.19 to 0.15) | 0.825 |
|  | 60 mo, imputed^b^ | -0.46 (1.03) | -0.49 (1.04) | -0.44 (1.03) | 0.810 | 0.02 (-0.14 to 0.18) | 0.772 | 0.05 (-0.10 to 0.21) | 0.507 | -0.03 (-0.19 to 0.13) | 0.728 |
|  | 60 mo, imputed^c^ | -0.45 (1.02) | -0.50 (1.02) | -0.43 (1.01) | 0.686 | 0.02 (-0.14 to 0.18) | 0.823 | 0.07 (-0.09 to 0.23) | 0.399 | -0.05 (-0.22 to 0.11) | 0.544 |

SP = sulfadoxine-pyrimethamine. AZI-SP = intervention group with monthly SP and two doses of azithromycin

^a^ Adjusted for maternal malaria at enrollment, HIV status, height, body mass index, number of previous pregnancies, number of school years, and child sex.

^b^ Multiple imputation for missing data by chained equations and 50 imputations. SD for multiple imputed data calculated as an average SD from 50 imputations.

^c^ Multiple imputation to replace weight measurements rounded to the full kilogram data by chained equations and 50 imputations. We considered all full kilogram weight measurements recorded at or after 36 months as censored within an interval of ± 0.5 kg of the full kilogram and used multiple imputation with interval censoring to replace the value. SD for multiple imputed data calculated as an average SD from 50 imputations.
