# Supplementary material for: The impact of maternal antenatal treatment with two doses of azithromycin and monthly sulphadoxine-pyrimethamine on child weight, mid-upper arm circumference and head circumference: A randomized controlled trial
Source: PLoS One. 2019 May 7;14(5):e0216536. doi: 10.1371/journal.pone.0216536 (PMC6504037; doi:10.1371/journal.pone.0216536)
Supplement: S4 Table — (DOCX) [file pone.0216536.s006.docx]

**S4 Table. Mean (SD) mid-upper arm-circumference (MUAC) and mid-upper arm circumference-for-age Z-score (MUACZ) by intervention group at one, six, 12, 24, 36, 48, and 60 months of age.**

| **Outcome** | **Age** | **Mean (SD)** | | | | **Comparison between AZI-SP and control group** | | **Comparison between AZI-SP and monthly SP group** | | **Comparison between monthly SP and control group** | |
| --- | --- | --- | --- | --- | --- | --- | --- | --- | --- | --- | --- |
|  |  | **Control** | **Monthly SP** | **AZI-SP** | **Overall p-value** | **Difference in means  (95% CI)** | **P-value** | **Difference in means  (95% CI)** | **P-value** | **Difference in means  (95% CI)** | **P-value** |
| Mean (SD) MUAC (cm) | 1 mo | 11.2 (0.9) | 11.3 (0.9) | 11.4 (0.9) | 0.027 | 0.2 (0.0 to 0.3) | 0.007 | 0.1 (0.0 to 0.2) | 0.181 | 0.1 (0.0 to 0.2) | 0.192 |
|  | 1 mo, adjusted^a^ | - | - | - | 0.151 | 0.1 (0.0 to 0.3) | 0.059 | 0.0 (-0.1 to 0.2) | 0.617 | 0.1 (0.0 to 0.2) | 0.186 |
|  | 1 mo, imputed^b^ | 11.2 (0.9) | 11.3 (0.9) | 11.4 (0.9) | 0.027 | 0.2 (0.0 to 0.3) | 0.007 | 0.1 (0.0 to 0.2) | 0.181 | 0.1 (0.0 to 0.2) | 0.192 |
|  | 6 mo | 13.3 (1.1) | 13.4 (1.2) | 13.5 (1.2) | 0.052 | 0.2 (0.0 to 0.4) | 0.016 | 0.1 (0.0 to 0.3) | 0.107 | 0.1 (-0.1 to 0.2) | 0.460 |
|  | 6 mo, adjusted^a^ | - | - | - | 0.183 | 0.2 (-0.0 to 0.3) | 0.070 | 0.1 (-0.1 to 0.2) | 0.515 | 0.1 (0.0 to 0.3) | 0.269 |
|  | 6 mo, imputed^b^ | 13.3 (1.1) | 13.3 (1.2) | 13.5 (1.2) | 0.094 | 0.2 (0.0 to 0.3) | 0.041 | 0.1 (0.0 to 0.3) | 0.089 | 0.0 (-0.1 to 0.2) | 0.760 |
|  | 12 mo | 13.6 (1.2) | 13.7 (1.2) | 13.7 (1.2) | 0.323 | 0.1 (0.0 to 0.3) | 0.135 | 0.1 (-0.1 to 0.2) | 0.540 | 0.1 (-0.1 to 0.3) | 0.367 |
|  | 12 mo, adjusted^a^ | - | - | - | 0.182 | 0.2 (0.0 to 0.3) | 0.099 | 0.0 (-0.2 to 0.2) | 0.855 | 0.1 (0.0 to 0.3) | 0.133 |
|  | 12 mo, imputed^b^ | 13.6 (1.2) | 13.7 (1.1) | 13.7 (1.2) | 0.468 | 0.1 (-0.1 to 0.3) | 0.218 | 0.1 (-0.1 to 0.2) | 0.494 | 0.0 (-0.1 to 0.2) | 0.570 |
|  | 24 mo | 14.1 (1.0) | 14.2 (1.1) | 14.3 (1.1) | 0.070 | 0.2 (0.0 to 0.4) | 0.021 | 0.1 (-0.1 to 0.3) | 0.237 | 0.1 (-0.1 to 0.3) | 0.286 |
|  | 24 mo, adjusted^a^ | - | - | - | 0.278 | 0.1 (0.0 to 0.3) | 0.110 | 0.1 (-0.1 to 0.2) | 0.455 | 0.1 (-0.1 to 0.2) | 0.388 |
|  | 24 mo, imputed^b^ | 14.1 (1.0) | 14.2 (1.1) | 14.3 (1.1) | 0.063 | 0.2 (0.0 to 0.3) | 0.021 | 0.1 (0.0 to 0.3) | 0.122 | 0.1 (-0.1 to 0.2) | 0.472 |
|  | 36 mo | 14.7 (1.1) | 14.8 (1.1) | 14.8 (1.1) | 0.338 | 0.1 (-0.1 to 0.3) | 0.209 | 0.0 (-0.2 to 0.2) | 0.933 | 0.1 (-0.1 to 0.3) | 0.190 |
|  | 36 mo, adjusted^a^ | - | - | - | 0.485 | 0.1 (-0.1 to 0.3) | 0.277 | 0.0 (-0.2 to 0.2) | 0.956 | 0.1 (-0.1 to 0.3) | 0.311 |
|  | 36 mo, imputed^b^ | 14.7 (1.1) | 14.8 (1.1) | 14.8 (1.1) | 0.388 | 0.1 (-0.1 to 0.2) | 0.295 | 0.0 (-0.2 to 0.1) | 0.800 | 0.1 (-0.1 to 0.3) | 0.194 |
|  | 48 mo | 15.0 (1.0) | 15.0 (1.0) | 15.0 (1.1) | 0.978 | 0.0 (-0.2 to 0.2) | 0.978 | 0.0 (-0.1 to 0.2) | 0.847 | 0.0 (-0.2 to 0.1) | 0.868 |
|  | 48 mo, adjusted^a^ | - | - | - | 0.994 | 0.0 (-0.2 to 0.2) | 0.922 | 0.0 (-0.2 to 0.2) | 0.987 | 0.0 (-0.2 to 0.2) | 0.934 |
|  | 48 mo, imputed^b^ | 15.0 (1.0) | 15.0 (1.1) | 15.0 (1.1) | 0.943 | 0.0 (-0.2 to 0.1) | 0.773 | 0.0 (-0.2 to 0.1) | 0.761 | 0.0 (-0.2 to 0.2) | 0.986 |
|  | 60 mo | 15.2 (1.0) | 15.2 (1.0) | 15.2 (1.1) | 0.951 | 0.0 (-0.1 to 0.2) | 0.803 | 0.0 (-0.1 to 0.2) | 0.766 | 0.0 (-0.2 to 0.2) | 0.956 |
|  | 60 mo, adjusted^a^ | - | - | - | 0.922 | 0.0 (-0.1 to 0.2) | 0.698 | 0.0 (-0.2 to 0.2) | 0.906 | 0.0 (-0.1 to 0.2) | 0.779 |
|  | 60 mo, imputed^b^ | 15.2 (1.0) | 15.2 (1.0) | 15.2 (1.1) | 0.946 | 0.0 (-0.1 to 0.2) | 0.910 | 0.0 (-0.2 to 0.1) | 0.842 | 0.0 (-0.1 to 0.2) | 0.743 |
| Mean (SD) MUACZ | 1 mo | NA | NA | NA | NA | NA | NA | NA | NA | NA | NA |
|  | 1 mo, adjusted^a^ | NA | NA | NA | NA | NA | NA | NA | NA | NA | NA |
|  | 1 mo, imputed^b^ | NA | NA | NA | NA | NA | NA | NA | NA | NA | NA |
|  | 6 mo | -0.71 (1.04) | -0.67 (1.12) | -0.51 (1.14) | 0.044 | 0.19 (0.04 to 0.35) | 0.016 | 0.15 (-0.01 to 0.32) | 0.068 | 0.04 (-0.12 to 0.20) | 0.606 |
|  | 6 mo, adjusted^a^ | - | - | - | 0.270 | 0.13 (-0.03 to 0.29) | 0.112 | 0.05 (-0.13 to 0.22) | 0.595 | 0.08 (-0.08 to 0.25) | 0.325 |
|  | 6 mo, imputed^b^ | -0.69 (1.04) | -0.69 (1.13) | -0.53 (1.14) | 0.080 | 0.16 (0.00 to 0.31) | 0.044 | 0.15 (-0.01 to 0.31) | 0.059 | 0.01 (-0.15 to 0.16) | 0.949 |
|  | 12 mo | -0.80 (1.08) | -0.74 (1.06) | -0.67 (1.12) | 0.300 | 0.13 (-0.03 to 0.29) | 0.121 | 0.07 (-0.09 to 0.23) | 0.422 | 0.06 (-0.10 to 0.22) | 0.440 |
|  | 12 mo, adjusted^a^ | - | - | - | 0.208 | 0.13 (-0.03 to 0.30) | 0.119 | 0.01 (-0.16 to 0.18) | 0.920 | 0.13 (-0.04 to 0.29) | 0.140 |
|  | 12 mo, imputed^b^ | -0.79 (1.07) | -0.75 (1.05) | -0.68 (1.12) | 0.423 | 0.10 (-0.05 to 0.26) | 0.197 | 0.07 (-0.09 to 0.22) | 0.384 | 0.03 (-0.12 to 0.19) | 0.657 |
|  | 24 mo | -0.86 (0.95) | -0.79 (1.01) | -0.68 (1.00) | 0.064 | 0.17 (0.03 to 0.32) | 0.020 | 0.11 (-0.04 to 0.26) | 0.147 | 0.06 (-0.09 to 0.21) | 0.407 |
|  | 24 mo, adjusted^a^ | - | - | - | 0.295 | 0.12 (-0.03 to 0.27) | 0.119 | 0.06 (-0.09 to 0.21) | 0.453 | 0.06 (-0.09 to 0.22) | 0.418 |
|  | 24 mo, imputed^b^ | -0.83 (0.94) | -0.80 (1.00) | -0.67 (1.01) | 0.057 | 0.17 (0.02 to 0.31) | 0.022 | 0.13 (-0.02 to 0.28) | 0.080 | 0.04 (-0.11 to 0.18) | 0.606 |
|  | 36 mo | -0.87 (0.93) | -0.78 (0.91) | -0.77 (0.91) | 0.344 | 0.09 (-0.05 to 0.23) | 0.190 | 0.00 (-0.13 to 0.14) | 0.961 | 0.09 (-0.05 to 0.23) | 0.214 |
|  | 36 mo, adjusted^a^ | - | - | - | 0.473 | 0.08 (-0.06 to 0.23) | 0.270 | 0.00 (-0.14 to 0.14) | 0.963 | 0.08 (-0.07 to 0.23) | 0.298 |
|  | 36 mo, imputed^b^ | -0.87 (0.91) | -0.78 (0.90) | -0.80 (0.93) | 0.400 | 0.07 (-0.06 to 0.21) | 0.290 | -0.01 (-0.15 to 0.12) | 0.842 | 0.09 (-0.05 to 0.22) | 0.205 |
|  | 48 mo | -0.93 (0.82) | -0.94 (0.83) | -0.93 (0.85) | 0.987 | 0.00 (-0.13 to 0.13) | 0.967 | 0.01 (-0.12 to 0.14) | 0.875 | -0.01 (-0.13 to 0.12) | 0.907 |
|  | 48 mo, adjusted^a^ | - | - | - | 0.993 | -0.01 (-0.14 to 0.13) | 0.909 | -0.01 (-0.14 to 0.13) | 0.940 | 0.00 (-0.13 to 0.13) | 0.968 |
|  | 48 mo, imputed^b^ | -0.93 (0.81) | -0.92 (0.83) | -0.95 (0.86) | 0.923 | -0.02 (-0.15 to 0.10) | 0.753 | -0.02 (-0.15 to 0.10) | 0.711 | 0.00 (-0.12 to 0.13) | 0.954 () |
|  | 60 mo | -1.09 (0.74) | -1.09 (0.76) | -1.08 (0.83) | 0.997 | 0.00 (-0.12 to 0.13) | 0.940 | 0.00 (-0.12 to 0.13) | 0.948 | 0.00 (-0.12 to 0.12) | 0.992 |
|  | 60 mo, adjusted^a^ | - | - |  | 0.9267 | 0.02 (-0.10 to 0.15) | 0.730 | 0.00 (-0.13 to 0.13) | 0.971 | 0.02 (-0.10 to 0.14) | 0.747 |
|  | 60 mo, imputed^b^ | -1.09 (0.75) | -1.07 (0.77) | -1.10 (0.84) | 0.903 | -0.01 (-0.13 to 0.11) | 0.896 | -0.03 (-0.15 to 0.09) | 0.664 | 0.02 (-0.10 to 0.13) | 0.750 |

SP = sulfadoxine-pyrimethamine. AZI-SP = intervention group with monthly SP and two doses of azithromycin. NA = Not applicable, MUACZ is available only from three months of age onwards.

^a^ Adjusted for maternal malaria at enrollment, HIV status, height, body mass index, number of previous pregnancies, number of school years, and child sex.

^b^ Multiple imputation for missing data by chained equations and 50 imputations. SD for multiple imputed data calculated as an average SD from 50 imputations.
