# Supplementary material for: The impact of maternal antenatal treatment with two doses of azithromycin and monthly sulphadoxine-pyrimethamine on child weight, mid-upper arm circumference and head circumference: A randomized controlled trial
Source: PLoS One. 2019 May 7;14(5):e0216536. doi: 10.1371/journal.pone.0216536 (PMC6504037; doi:10.1371/journal.pone.0216536)
Supplement: S5 Table — (DOCX) [file pone.0216536.s007.docx]

**S5 Table. Mean (SD) head circumference (HC) and head circumference-for-age Z-score (HCZ) by intervention group at one, six, 12, 24, 36, 48, and 60 months of age.**

| **Outcome** | **Age** | **Mean (SD)** | | | | **Comparison between AZI-SP and control group** | | **Comparison between AZI-SP and monthly SP group** | | **Comparison between monthly SP and control group** | |
| --- | --- | --- | --- | --- | --- | --- | --- | --- | --- | --- | --- |
|  |  | **Control** | **Monthly SP** | **AZI-SP** | **Overall p-value** | **Difference in means  (95% CI)** | **P-value** | **Difference in means  (95% CI)** | **P-value** | **Difference in means  (95% CI)** | **P-value** |
| Mean (SD) HC (cm) | 1 mo | 37.1 (1.5) | 37.2 (1.5) | 37.4 (1.3) | 0.013 | 0.3 (0.1 to 0.5) | 0.004 | 0.2 (0.0 to 0.4) | 0.066 | 0.1 (-0.1 to 0.3) | 0.335 |
|  | 1 mo, adjusted^a^ | - | - | - | 0.028 | 0.2 (0.0 to 0.4) | 0.018 | 0.2 (0.0 to 0.4) | 0.028 | 0.0 (-0.2 to 0.2) | 0.889 |
|  | 1 mo, imputed^b^ | 37.1 (1.5) | 37.2 (1.5) | 37.4 (1.3) | 0.012 | 0.3 (0.1 to 0.5) | 0.004 | 0.2 (0.0 to 0.4) | 0.054 | 0.1 (-0.1 to 0.3) | 0.362 |
|  | 6 mo | 43.1 (1.6) | 43.0 (1.5) | 43.3 (1.5) | 0.015 | 0.2 (0.0 to 0.4) | 0.072 | 0.3 (0.1 to 0.5) | 0.004 | -0.1 (-0.3 to 0.1) | 0.354 |
|  | 6 mo, adjusted^a^ | - | - | - | 0.001 | 0.2 (0.0 to 0.4) | 0.048 | 0.4 (0.2 to 0.6) | <0.001 | -0.2 (-0.4 to 0.0) | 0.108 |
|  | 6 mo, imputed^b^ | 43.1 (1.6) | 43.0 (1.5) | 43.3 (1.5) | 0.012 | 0.2 (0.0 to 0.4) | 0.065 | 0.3 (0.1 to 0.5) | 0.003 | -0.1 (-0.3 to 0.1) | 0.329 |
|  | 12 mo | 45.3 (1.5) | 45.4 (1.6) | 45.4 (1.4) | 0.551 | 0.1 (-0.1 to 0.3) | 0.276 | 0.1 (-0.2 to 0.3) | 0.594 | 0.1 (-0.2 to 0.3) | 0.625 |
|  | 12 mo, adjusted^a^ | - | - | - | 0.440 | 0.1 (-0.1 to 0.4) | 0.226 | 0.1 (-0.1 to 0.3) | 0.334 | 0.0 (-0.2 to 0.2) | 0.827 |
|  | 12 mo, imputed^b^ | 45.3 (1.5) | 45.3 (1.6) | 45.4 (1.5) | 0.587 | 0.1 (-0.1 to 0.3) | 0.303 | 0.1 (-0.2 to 0.3) | 0.632 | 0.1 (-0.2 to 0.3) | 0.618 |
|  | 24 mo | 47.4 (1.4) | 47.4 (1.6) | 47.5 (1.5) | 0.476 | 0.1 (-0.1 to 0.6) | 0.226 | 0.1 (-0.1 to 0.3) | 0.481 | 0.1 (-0.2 to 0.3) | 0.644 |
|  | 24 mo, adjusted^a^ | - | - | - | 0.329 | 0.1 (-0.1 to 0.3) | 0.313 | 0.2 (-0.1 to 0.4) | 0.145 | -0.1 (-0.3 to 0.2) | 0.630 |
|  | 24 mo, imputed^b^ | 47.3 (1.4) | 47.4 (1.5) | 47.5 (1.4) | 0.158 | 0.2 (0.0 to 0.4) | 0.072 | 0.2 (-0.1 to 0.4) | 0.147 | 0.0 (-0.2 to 0.2) | 0.775 |
|  | 36 mo | 48.4 (1.4) | 48.4 (1.5) | 48.5 (1.5) | 0.744 | 0.1 (-0.1 to 0.3) | 0.466 | 0.1 (-0.2 to 0.3) | 0.576 | 0.0 (-0.2 to 0.2) | 0.882 |
|  | 36 mo, adjusted^a^ | - | - | - | 0.344 | 0.0 (-0.2 to 0.3) | 0.674 | 0.2 (-0.1 to 0.4) | 0.156 | -0.1 (-0.3 to 0.1) | 0.310 |
|  | 36 mo, imputed^b^ | 48.4 (1.4) | 48.3 (1.5) | 48.5 (1.4) | 0.585 | 0.1 (-0.1 to 0.3) | 0.424 | 0.1 (-0.1 to 0.3) | 0.335 | 0.0 (-0.2 to 0.2) | 0.853 |
|  | 48 mo | 49.1 (1.4) | 49.0 (1.5) | 49.2 (1.4) | 0.287 | 0.1 (-0.1 to 0.3) | 0.365 | 0.2 (0.0 to 0.4) | 0.117 | -0.1 (-0.3 to 0.1) | 0.495 |
|  | 48 mo, adjusted^a^ | - | - | - | 0.119 | 0.1 (-0.1 to 0.3) | 0.525 | 0.2 (0.0 to 0.4) | 0.043 | -0.2 (-0.4 to 0.1) | 0.165 |
|  | 48 mo, imputed^b^ | 49.0 (1.4) | 49.0 (1.5) | 49.2 (1.4) | 0.272 | 0.1 (-0.1 to 0.3) | 0.263 | 0.2 (0.0 to 0.4) | 0.122 | 0.0 (-0.3 to 0.2) | 0.650 |
|  | 60 mo | 49.3 (1.5) | 49.4 (1.5) | 49.5 (1.5) | 0.248 | 0.2 (0.0 to 0.4) | 0.095 | 0.1 (-0.1 to 0.3) | 0.396 | 0.1 (-0.1 to 0.3) | 0.440 |
|  | 60 mo, adjusted^a^ | - | - | - | 0.302 | 0.1 (-0.1 to 0.4) | 0.215 | 0.2 (-0.1 to 0.4) | 0.154 | 0.0 (-0.3 to 0.2) | 0.843 |
|  | 60 mo, imputed^b^ | 49.3 (1.5) | 49.4 (1.5) | 49.5 (1.5) | 0.314 | 0.2 (-0.1 to 0.4) | 0.137 | 0.1 (-0.1 to 0.3) | 0.303 | 0.0 (-0.2 to 0.3) | 0.681 |
| Mean (SD) HCZ | 1 mo | 0.03 (1.24) | 0.08 (1.21) | 0.33 (1.10) | <0.001 | 0.30 (0.14 to 0.46) | <0.001 | 0.25 (0.09 to 0.41) | 0.003 | 0.05 (-0.12 to 0.22) | 0.544 |
|  | 1 mo, adjusted^a^ | - | - | - | 0.004 | 0.24 (0.07 to 0.41) | 0.005 | 0.24 (0.07 to 0.41) | 0.005 | 0.00 (-0.17 to 0.18) | 0.966 |
|  | 1 mo, imputed^b^ | 0.03 (1.24) | 0.08 (1.21) | 0.33 (1.10) | <0.001 | 0.30 (0.14 to 0.46) | <0.001 | 0.25 (0.09 to 0.41) | 0.002 | 0.05 (-0.12 to 0.22) | 0.543 |
|  | 6 mo | 0.18 (1.20) | 0.07 (1.07) | 0.36 (1.12) | 0.001 | 0.18 (0.01 to 0.35) | 0.037 | 0.29 (0.13 to 0.45) | <0.001 | -0.11 (-0.28 to 0.05) | 0.178 |
|  | 6 mo, adjusted^a^ | - | - | - | 0.001 | 0.17 (0.01 to 0.34) | 0.065 | 0.31 (0.15 to 0.47) | <0.001 | -0.14 (-0.32 to 0.03) | 0.101 |
|  | 6 mo, imputed^b^ | 0.18 (1.18) | 0.07 (1.07) | 0.35 (1.11) | 0.001 | 0.17 (0.01 to 0.33) | 0.038 | 0.28 (0.13 to 0.43) | <0.001 | -0.11 (-0.27 to 0.05) | 0.163 |
|  | 12 mo | -0.17 (1.05) | -0.15 (1.15) | -0.06 (1.08) | 0.334 | 0.11 (-0.04 to 0.27) | 0.158 | 0.09 (-0.08 to 0.25) | 0.287 | 0.02 (-0.14 to 0.19) | 0.775 |
|  | 12 mo, adjusted^a^ | - | - | - | 0.449 | 0.10 (-0.06 to 0.27) | 0.228 | 0.08 (-0.09 to 0.25) | 0.354 | 0.02 (-0.15 to 0.19) | 0.798 |
|  | 12 mo, imputed^b^ | -0.17 (1.04) | -0.15 (1.13) | -0.07 (1.09) | 0.364 | 0.10 (-0.05 to 0.26) | 0.176 | 0.08 (-0.07 to 0.24) | 0.296 | 0.02 (-0.14 to 0.18) | 0.794 |
|  | 24 mo | -0.28 (0.95) | -0.28 (1.05) | -0.17 (1.01) | 0.241 | 0.11 (-0.03 to 0.26) | 0.133 | 0.11 (-0.04 to 0.26) | 0.158 | 0.00 (-0.15 to 0.15) | 0.988 |
|  | 24 mo, adjusted^a^ | - | - | - | 0.321 | 0.08 (-0.08 to 0.23) | 0.324 | 0.12 (-0.04 to 0.28) | 0.139 | -0.04 (-0.20 to 0.11) | 0.597 |
|  | 24 mo, imputed^b^ | -0.29 (0.94) | -0.29 (1.03) | -0.14 (0.99) | 0.051 | 0.15 (0.01 to 0.29) | 0.033 | 0.15 (0.01 to 0.30) | 0.037 | 0.00 (-0.15 to 0.14) | 0.958 |
|  | 36 mo | -0.43 (0.93) | -0.46 (1.00) | -0.37 (0.98) | 0.450 | 0.07 (-0.07 to 0.21) | 0.349 | 0.09 (-0.06 to 0.24) | 0.231 | -0.02 (-0.17 to 0.13) | 0.770 |
|  | 36 mo, adjusted^a^ | - | - | - | 0.334 | 0.03 (-0.12 to 0.19) | 0.668 | 0.11 (-0.04 to 0.27) | 0.150 | -0.08 (-0.23 to 0.07) | 0.305 |
|  | 36 mo, imputed^b^ | -0.44 (0.93) | -0.48 (0.98) | -0.37 (0.97) | 0.316 | 0.07 (-0.06 to 0.21) | 0.289 | 0.11 to (-0.04 to 0.25) | 0.140 | -0.03 (-0.17 to 0.11) | 0.653 |
|  | 48 mo | -0.51 (0.91) | -0.58 (0.97) | -0.43 (0.90) | 0.125 | 0.08 (-0.06 to 0.22) | 0.260 | 0.15 (0.01 to 0.29) | 0.042 | -0.07 (-0.21 to 0.08) | 0.346 |
|  | 48 mo, adjusted^a^ | - | - | - | 0.113 | 0.05 (-0.10 to 0.19) | 0.523 | 0.16 (0.01 to 0.30) | 0.041 | -0.11 (-0.26 to 0.04) | 0.159 |
|  | 48 mo, imputed^b^ | -0.51 (0.91) | -0.56 (0.97) | -0.42 (0.91) | 0.101 | 0.10 (-0.04 to 0.23) | 0.160 | 0.15 (0.01 to 0.28) | 0.037 | -0.05 (-0.19 to 0.09) | 0.472 to |
|  | 60 mo | -0.72 (0.95) | -0.67 (0.98) | -0.56 (0.98) | 0.121 | 0.15 (0.00 to 0.31) | 0.044 | 0.11 (-0.05 to 0.26) | 0.173 | 0.05 (-0.11 to 0.20) | 0.542 |
|  | 60 mo, adjusted^a^ | - | - | - | 0.273 | 0.11 (-0.05 to 0.27) | 0.184 | 0.12 (-0.04 to 0.28) | 0.144 | -0.01 (-0.17 to 0.15) | 0.888 |
|  | 60 mo, imputed^b^ | -0.71 (0.95) | -0.69 (0.98) | -0.58 (0.97) | 0.173 | 0.13 (-0.01 to 0.27) | 0.079 | 0.11 (-0.04 to 0.25) | 0.143 | 0.02 (-0.13 to 0.16) | 0.809 |

SP = sulfadoxine-pyrimethamine. AZI-SP = intervention group with monthly SP and two doses of azithromycin

^a^ Adjusted for maternal malaria at enrollment, HIV status, height, body mass index, number of previous pregnancies, number of school years, and child sex.

^b^ Multiple imputation for missing data by chained equations and 50 imputations. SD for multiple imputed data calculated as an average SD from 50 imputations.
