# Supplementary material for: The impact of maternal antenatal treatment with two doses of azithromycin and monthly sulphadoxine-pyrimethamine on child weight, mid-upper arm circumference and head circumference: A randomized controlled trial
Source: PLoS One. 2019 May 7;14(5):e0216536. doi: 10.1371/journal.pone.0216536 (PMC6504037; doi:10.1371/journal.pone.0216536)
Supplement: S6 Table — Underweight defined as weight-for-age Z-score (WAZ) < -2. Wasting defined as weight-for-height Z-score (WHZ) < -2. Low mid-upper arm circumference (MUAC) defined MUAC-for-age Z-score (MUACZ) < -2. Small head circumference (HC) defined as HC-for-age Z-score < -2. (DOCX) [file pone.0216536.s008.docx]

**S6 Table.** **Prevalence of underweight, wasting, low mid-upper arm-circumference, and small head circumference by intervention group at one, six, 12, 24, 36, 48, and 60 months of age.**

Underweight defined as weight-for-age Z-score (WAZ) < -2. Wasting defined as weight-for-height Z-score (WHZ) < -2. Low mid-upper arm circumference (MUAC) defined MUAC-for-age Z-score (MUACZ) < -2. Small head circumference (HC) defined as HC-for-age Z-score < -2.

| **Outcome** | **Age** | **% of infants with outcome (n/N)** | | | | **Comparison between AZI-SP and control group** | | **Comparison between AZI-SP and monthly SP group** | | **Comparison between monthly SP and control group** | |
| --- | --- | --- | --- | --- | --- | --- | --- | --- | --- | --- | --- |
|  |  | **Control** | **Monthly SP** | **AZI-SP** | **Overall p-value** | **Risk ratio (95% CI)** | **P-value** | **Risk ratio (95% CI)** | **P-value** | **Risk ratio (95% CI)** | **P-value** |
| Underweight | 1 mo | 12.7% (51/402) | 10.4% (41/394) | 6.7% (27/401) | 0.022 | 0.53 (0.34 to 0.83) | 0.006 | 0.65 (0.40 to 1.03) | 0.069 | 0.82 (0.55 to 1.22) | 0.328 |
|  | 1 mo, adjusted^a^ | - | - | - | 0.132 | 0.65 (0.42 to 1.02) | 0.060 | 0.86 (0.53 to 1.39) | 0.533 | 0.76 (0.51 to 1.14) | 0.181 |
|  | 1 mo, imputed^b^ | 12.6% | 10.5% | 6.8% | 0.025 | 0.54 (0.34 to 0.84) | 0.007 | 0.64 (0.40 to 1.02) | 0.062 | 0.84 (0.56 to 1.24) | 0.373 |
|  | 6 mo | 13.8% (50/363) | 14.0% (51/364) | 9.1% (34/374) | 0.078 | 0.66 (0.44 to 1.00) | 0.048 | 0.65 (0.43 to 0.98) | 0.038 | 1.02 (0.71 to 1.46) | 0.926 |
|  | 6 mo, adjusted^a^ | - | - | - | 0.235 | 0.70 (0.46 to 1.06) | 0.095 | 0.76 (0.49 to 1.16) | 0.197 | 0.93 (0.64 to 1.35) | 0.700 |
|  | 6 mo, imputed^b^ | 13.5% | 14.4% | 9.4% | 0.087 | 0.69 (0.46 to 1.04) | 0.077 | 0.65 (0.44 to 0.97) | 0.033 | 1.07 (0.75 to 1.52) | 0.725 |
|  | 12 mo | 16.6% (57/343) | 15.9% (55/347) | 16.3% (59/362) | 0.964 | 0.98 (0.70 to 1.37) | 0.909 | 1.03 (0.73 to 1.44) | 0.872 | 0.95 (0.68 to 1.34) | 0.786 |
|  | 12 mo, adjusted^a^ | - | - | - | 0.619 | 0.97 (0.69 to 1.35) | 0.850 | 1.14 (0.80 to 1.63) | 0.464 | 0.85 (0.60 to 1.19) | 0.346 |
|  | 12 mo, imputed^b^ | 16.4% | 15.8% | 16.3% | 0.972 | 1.00 (0.72 to 1.38) | 0.982 | 1.03 (0.74 to 1.43) | 0.843 | 0.96 (0.69 to 1.34) | 0.829 |
|  | 24 mo | 24.2% (81/335) | 24.7% (85/344) | 21.1% (73/346) | 0.489 | 0.87 (0.66 to 1.16) | 0.342 | 0.85 (0.65 to 1.12) | 0.261 | 1.02 (0.78 to 1.34) | 0.874 |
|  | 24 mo, adjusted^a^ | - | - | - | 0.739^c^ | 0.90 (0.68 to 1.20) | 0.482 | 0.91 0.69 to 1.20 | 0.510 | 0.99 (0.76 to 1.30) | 0.963 |
|  | 24 mo, imputed^b^ | 23.8% | 24.4% | 20.4% | 0.378 | 0.86 (0.65 to 1.13) | 0.273 | 0.83 (0.63 to 1.09) | 0.185 | 1.03 (0.79 to 1.34) | 0.838 |
|  | 36 mo | 23.9% (78/326) | 23.6% (78/331) | 21.1% (74/351) | 0.634 | 0.88 (0.66 to 1.17) | 0.381 | 0.89 (0.68 to 1.18) | 0.436 | 0.98 (0.75 to 1.30) | 0.914 |
|  | 36 mo, adjusted^a^ | - | - | - | 0.855 | 0.93 (0.70 to 1.25) | 0.643 | 0.93 (0.70 to 1.23) | 0.609 | 1.01 (0.76 to 1.33) | 0.967 |
|  | 36 mo, imputed^b^ | 23.8% | 23.6% | 21.2% | 0.681 | 0.89 (0.68 to 1.18) | 0.426 | 0.90 (0.68 to 1.19) | 0.460 | 0.99 (0.76 to 1.31) | 0.962 |
|  | 36 mo, imputed^d^ | 23.7% | 23.5% | 21.4% | 0.746 | 0.90 (0.68 to 1.20) | 0.492 | 0.91 (0.69 to 1.21) | 0.523 | 0.99 (0.75 to 1.31) | 0.954 |
|  | 48 mo | 22.2% (73/329) | 23.4% (76/325) | 22.4% (74/331) | 0.925 | 1.01 (0.76 to 1.34) | 0.959 | 0.96 (0.72 to 1.27) | 0.754 | 1.05 (0.79 to 1.40) | 0.718 |
|  | 48 mo, adjusted^a^ | - | - | - | 0.898^c^ | 1.05 (0.78 to 1.40) | 0.764 | 0.98 (0.74 to 1.30) | 0.885 | 1.07 (0.81 to 1.41) | 0.647 |
|  | 48 mo, imputed^b^ | 21.6% | 23.6% | 22.6% | 0.822 | 1.04 (0.78 to 1.39) | 0.770 | 0.95 (0.73 to 1.25) | 0.733 | 1.09 (0.83 to 1.45) | 0.533 |
|  | 48 mo, imputed^d^ | 22.1% | 22.8% | 22.2% | 0.973 | 1.01 (0.75 to 1.35) | 0.966 | 0.97 (0.73 to 1.30) | 0.858 | 1.03 (0.77 to 1.39) | 0.827 |
|  | 60 mo | 20.1% (63/314) | 19.4% (60/309) | 16.5% (53/321) | 0.479 | 0.82 (0.59 to 1.15) | 0.250 | 0.85 (0.61 to 1.19) | 0.342 | 0.97 (0.70 to 1.33) | 0.840 |
|  | 60 mo, adjusted^a^ | - | - | - | 0.543 | 0.82 (0.57 to 1.17) | 0.279 | 0.91 (0.63 to 1.33) | 0.629 | 0.90 (0.65 to 1.24) | 0.519 |
|  | 60 mo, imputed^b^ | 19.8% | 19.5% | 17.5% | 0.708 | 0.88 (0.64 to 1.22) | 0.443 | 0.90 (0.65 to 1.23) | 0.499 | 0.98 (0.72 to 1.34) | 0.923 |
|  | 60 mo, imputed^d^ | 19.5% | 17.7% | 15.4% | 0.432 | 0.79 (0.55 to 1.13) | 0.195 | 0.87 (0.60 to 1.25) | 0.441 | 0.91 (0.64 to 1.29) | 0.599 |
| Wasting | 1 mo | 1.8% (7/399) | 1.8% (7/391) | 1.8% (7/398) | 0.999 | 1.00 (0.35 to 2.83) | 0.996 | 0.98 (0.35 to 2.78) | 0.973 | 1.02 (0.36 to 2.88) | 0.970 |
|  | 1 mo, adjusted^a^ | - | - | - | 0.995 | 1.06 (0.33 to 3.38) | 0.921 | 1.04 (0.33 to 3.33) | 0.942 | 1.02 (0.32 to 3.19) | 0.978 |
|  | 1 mo, imputed^b^ | 1.7% | 1.8% | 1.8% | 0.999 | 1.01 (0.36 to 2.84) | 0.991 | 0.99 (0.35 to 2.79) | 0.981 | 1.02 (0.36 to 2.88) | 0.972 |
|  | 6 mo | 2.2% (8/358) | 3.6% (13/360) | 2.7% (10/371) | 0.533 | 1.21 (0.48 to 3.02) | 0.689 | 0.75 (0.33 to 1.68) | 0.480 | 1.62 (0.68 to 3.85) | 0.279 |
|  | 6 mo, adjusted^a^ | - | - | - | 0.544 | 1.15 (0.45 to 2.91) | 0.770 | 0.73 (0.32 to 1.66) | 0.446 | 1.58 (0.66 to 3.78) | 0.301 |
|  | 6 mo, imputed^b^ | 2.3% | 4.1% | 2.7% | 0.367 | 1.16 (0.46 to 2.91) | 0.754 | 0.66 (0.30 to 1.47) | 0.310 | 1.75 (0.75 to 4.11) | 0.197 |
|  | 12 mo | 4.7% (16/341) | 5.2% (18/347) | 4.4% (16/362) | 0.889 | 0.94 (0.48 to 1.85) | 0.863 | 0.85 (0.44 to 1.64) | 0.633 | 1.11 (0.57 to 2.13) | 0.765 |
|  | 12 mo, adjusted^a^ | - | - | - | 0.913 | 0.86 (0.42 to 1.75) | 0.678 | 0.95 (0.46 to 1.96) | 0.888 to | 0.91 (0.45 to 1.81) | 0.782 |
|  | 12 mo, imputed^b^ | 4.6% | 5.1% | 4.5% | 0.904 | 0.98 (0.50 to 1.91) | 0.949 | 0.87 (0.46 to 1.66) | 0.679 | 1.12 (0.59 to 2.14) | 0.729 |
|  | 24 mo | 5.4% (18/335) | 5.3% (18/343) | 4.9% (17/346) | 0.962 | 0.91 (0.48 to 1.74) | 0.786 | 0.94 (0.49 to 1.78) | 0.841 | 0.98 (0.52 to 1.84) | 0.942 |
|  | 24 mo, adjusted^a^ | - | - | - | 0.979 | 0.93 (0.47 to 1.83) | 0.836 | 0.96 (0.49 to 1.89) | 0.913 | 0.97 (0.51 to 1.85) | 0.919 |
|  | 24 mo, imputed^b^ | 5.0% | 5.1% | 4.4% | 0.874 | 0.87 (0.45 to 1.68) | 0.672 | 0.85 (0.44 to 1.64) | 0.633 | 1.02 (0.54 to 1.91) | 0.960 |
|  | 36 mo | 7.1% (23/325) | 7.0% (23/331) | 5.1% (18/351) | 0.515 | 0.72 (0.39 to 1.33) | 0.300 | 0.74 (0.41 to 1.34) | 0.320 | 0.98 (0.56 to 1.73) | 0.950 |
|  | 36 mo, adjusted^a^ | - | - | - | 0.677 | 0.79 (0.42 to 1.49) | 0.467 | 0.77 (0.41 to 1.43) | 0.411 | 1.03 (0.57 to 1.85) | 0.933 |
|  | 36 mo, imputed^b^ | 7.3% | 6.6% | 5.3% | 0.568 | 0.73 (0.41 to 1.31) | 0.291 | 0.80 (0.45 to 1.45) | 0.467 | 0.91 (0.52 to 1.58) | 0.740 |
|  | 36 mo, imputed^d^ | 6.5% | 7.1% | 4.6% | 0.389 | 0.71 (0.36 to 1.38) | 0.310 | 0.65 (0.34 to 1.22) | 0.179 | 1.09 (0.60 to 1.99) | 0.774 |
|  | 48 mo | 5.2% (17/329) | 7.7% (25/325) | 6.7% (22/331) | 0.450 | 1.29 (0.68 to 2.42) | 0.436 | 0.86 (0.50 to 1.50) | 0.604 | 1.49 (0.80 to 2.76) | 0.206 |
|  | 48 mo, adjusted^a^ | - | - | - | 0.331 | 1.29 (0.66 to 2.52) | 0.449 | 0.81 (0.46 to 1.43) | 0.475 | 1.59 (0.86 to 2.94) | 0.140 |
|  | 48 mo, imputed^b^ | 5.3% | 8.2% | 7.1% | 0.361 | 1.33 (0.71 to 2.49) | 0.372 | 0.86 (0.51 to 1.46) | 0.574 | 1.55 (0.86 to 2.80) | 0.148 |
|  | 48 mo, imputed^d^ | 5.2% | 7.2% | 6.3% | 0.613 | 1.22 (0.63 to 2.39) | 0.553 | 0.88 (0.48 to 1.59) | 0.672 | 1.39 (0.72 to 2.69) | 0.326 |
|  | 60 mo | 6.8% (21/307) | 6.2% (19/305) | 6.3% (20/318) | 0.944 | 0.92 (0.51 to 1.66) | 0.781 | 1.01 (0.55 to 1.86) | 0.975 | 0.91 (0.50 to 1.66) | 0.760 |
|  | 60 mo, adjusted^a^ | - | - | - | 0.462 | 0.76 (0.40 to 1.46) | 0.416 | 1.12 (0.54 to 2.32) | 0.758 | 0.68 (0.36 to 1.29) | 0.241 |
|  | 60 mo, imputed^b^ | 7.1% | 6.8% | 6.4% | 0.943 | 0.90 (0.51 to 1.60) | 0.731 | 0.94 (0.53 to 1.68) | 0.839 | 0.96 (0.53 to 1.72) | 0.892 |
|  | 60 mo, imputed^d^ | 6.5% | 6.2% | 6.5% | 0.990 | 1.00 (0.53 to 1.88) | 0.990 | 1.04 (0.55 to 1.95) | 0.907 | 0.96 (0.50 to 1.82) | 0.899 |
| Low MUAC | 1 mo | NA | NA | NA | NA | NA | NA | NA | NA | NA | NA |
|  | 1 mo, adjusted^a^ | NA | NA | NA | NA | NA | NA | NA | NA | NA | NA |
|  | 1 mo, imputed^b^ | NA | NA | NA | NA | NA | NA | NA | NA | NA | NA |
|  | 6 mo | 11.1% (40/362) | 10.2% (37/364) | 8.0% (30/375) | 0.360 | 0.72 (0.46 to 1.14) | 0.161 | 0.79 (0.50 to 1.25) | 0.307 | 0.92 (0.60 to 1.40) | 0.699 |
|  | 6 mo, adjusted^a^ | - | - | - | 0.653 | 0.81 (0.51 to 1.28) | 0.370 | 0.92 (0.57 to 1.50) | 0.744 | 0.88 (0.56 to 1.37) | 0.564 |
|  | 6 mo, imputed^b^ | 10.8% | 10.7% | 8.3% | 0.440 | 0.77 (0.49 to 1.20) | 0.249 | 0.78 (0.50 to 1.21) | 0.265 to | 0.99 (0.65 to 1.50) | 0.961 |
|  | 12 mo | 11.6% (40/344) | 13.8% (48/349) | 12.4% (45/362) | 0.700 | 1.07 (0.72 to 1.59) | 0.743 | 0.90 (0.62 to 1.33) | 0.605 | 1.18 (0.80 to 1.76) | 0.405 |
|  | 12 mo, adjusted^a^ | - | - | - | 0.962 | 0.99 (0.66 to 1.49) | 0.958 | 0.95 (0.62 to 1.43) | 0.794 | 1.05 (0.69 to 1.58) | 0.832 |
|  | 12 mo, imputed^b^ | 11.2% | 13.5% | 12.6% | 0.643 | 1.12 (0.76 to 1.66) | 0.568 | 0.93 (0.64 to 1.35) | 0.703 | 1.20 (0.81 to 1.78) | 0.352 |
|  | 24 mo | 11.0% (37/337) | 11.4% (40/350) | 8.8% (31/352) | 0.484 | 0.80 (0.51 to 1.27) | 0.346 | 0.77 (0.49 to 1.20) | 0.251 | 1.04 (0.68 to 1.59) | 0.854 |
|  | 24 mo, adjusted^a^ | - | - | - | 0.571 | 0.82 (0.50 to 1.32) | 0.409 | 0.78 (0.48 to 1.26) | 0.309 | 1.05 (0.67 to 1.63) | 0.845 |
|  | 24 mo, imputed^b^ | 10.5% | 11.2% | 8.8% | 0.542 | 0.84 (0.53 to 1.32) | 0.451 | 0.78 (0.50 to 1.21) | 0.274 | 1.07 (0.70 to 1.65) | 0.750 |
|  | 36 mo | 10.3% (34/330) | 9.0% (30/335) | 9.0% (32/354) | 0.806 | 0.88 (0.55 to 1.40) | 0.581 | 1.01 (0.63 to 1.62) | 0.969 | 0.87 (0.54 to 1.39) | 0.561 |
|  | 36 mo, adjusted^a^ | - | - | - | 0.916 | 0.94 (0.58 to 1.52) | 0.803 | 1.04 (0.63 to 1.72) | 0.876 | 0.90 (0.56 to 1.46) | 0.680 |
|  | 36 mo, imputed^b^ | 10.1% | 8.8% | 9.4% | 0.857 | 0.94 (0.59 to 1.48) | 0.778 | 1.07 (0.67 to 1.71) | 0.781 | 0.88 (0.55 to 1.39) | 0.575 |
|  | 48 mo | 10.3% (34/331) | 10.0% (33/330) | 10.8% (36/334) | 0.946 | 1.05 (0.67 to 1.65) | 0.834 | 1.08 (0.69 to 1.69) | 0.743 | 0.97 (0.61 to 1.54) | 0.909 |
|  | 48 mo, adjusted^a^ | - | - | - | 0.898 | 1.11 (0.69 to 1.77) | 0.674 | 1.10 (0.69 to 1.75) | 0.700 | 1.01 (0.63 to 1.60) | 0.972 |
|  | 48 mo, imputed^b^ | 9.8% | 9.8% | 11.2% | 0.777 | 1.14 (0.73 to 1.78) | 0.553 to | 1.15 (0.74 to 1.77) | 0.541 | 1.00 (0.63 to 1.57) | 0.996 |
|  | 60 mo | 9.4% (29/308) | 9.4% (29/310) | 11.2% (36/322) | 0.687 | 1.19 (0.74 to 1.90) | 0.475 | 1.20 (0.75 to 1.90) | 0.451 | 0.99 (0.60 to 1.63) | 0.980 |
|  | 60 mo, adjusted^a^ | - | - | - | 0.812 | 1.15 (0.70 to 1.88) | 0.574 | 1.15 (0.70 to 1.88) | 0.585 | 1.00 (0.60 to 1.66) | 0.990 |
|  | 60 mo, imputed^b^ | 9.9% | 9.6% | 12.2% | 0.504 | 1.22 (0.78 to 1.91) | 0.374 | 1.27 (0.82 to 1.97) | 0.288 | 0.96 (0.60 to 1.54) | 0.879 |
| Small HC | 1 mo | 4.0% (16/403) | 3.8% (15/394) | 2.0% (8/401) | 0.232 | 0.50 (0.22 to 1.16) | 0.107 | 0.52 (0.22 to 1.22) | 0.135 | 0.96 (0.48 to 1.91) | 0.905 |
|  | 1 mo, adjusted^a^ | - | - | - | 0.367 | 0.56 (0.24 to 1.28) | 0.169 | 0.59 (0.24 to 1.44) | 0.250 | 0.94 (0.46 to 1.92) | 0.860 |
|  | 1 mo, imputed^b^ | 3.9% | 3.9% | 2.0% | 0.214 | 0.50 (0.22 to 1.16) | 0.107 | 0.50 (0.22 to 1.16) | 0.108 | 1.00 (0.50 to 1.98) | 0.996 |
|  | 6 mo | 2.2% (8/363) | 2.5% (9/362) | 1.6% (6/372) | 0.704 | 0.73 (0.26 to 2.09) | 0.560 | 0.65 (0.23 to 1.81) | 0.407 | 1.13 (0.44 to 2.89) | 0.802 |
|  | 6 mo, adjusted^a^ | - | - | - | 0.831 | 0.79 (0.26 to 2.35) | 0.669 | 0.71 (0.23 to 2.15) | 0.545 | 1.11 (0.42 to 2.92) | 0.834 |
|  | 6 mo, imputed^b^ | 2.1% | 2.5% | 1.7% | 0.749 | 0.83 (0.30 to 2.33) | 0.724 | 0.68 (0.25 to 1.87) | 0.458 | 1.22 (0.48 to 3.11) | 0.680 |
|  | 12 mo | 3.5% (12/340) | 5.2% (18/349) | 3.6% (13/359) | 0.480 | 1.03 (0.47 to 2.22) | 0.948 | 0.70 (0.35 to 1.41) | 0.321 | 1.46 (0.71 to 2.99) | 0.299 |
|  | 12 mo, adjusted^a^ | - | - | - | 0.577 | 1.00 (0.44 to 2.27) | 0.991 | 0.72 (0.34 to 1.52) | 0.384 | 1.40 (0.66 to 2.95) | 0.376 |
|  | 12 mo, imputed^b^ | 3.4% | 5.2% | 3.7% | 0.449 | 1.08 (0.50 to 2.31) | 0.845 | 0.72 (0.36 to 1.42) | 0.337 | 1.51 (0.75 to 3.03) | 0.250 |
|  | 24 mo | 2.4% (8/338) | 5.2% (18/349) | 4.0% (14/351) | 0.176 | 1.69 (0.72 to 3.97) | 0.232 | 0.77 (0.39 to 1.53) | 0.461 | 2.18 (0.96 to 4.95) | 0.063 |
|  | 24 mo, adjusted^a^ | - | - | - | 0.151 | 1.87 (0.77 to 4.56) | 0.169 | 0.83 (0.40 to 1.69) | 0.605 | 2.26 (0.99 to 5.15) | 0.053 |
|  | 24 mo, imputed^b^ | 2.4% | 4.9% | 3.6% | 0.213 | 1.51 (0.65 to 3.50) | 0.338 | 0.74 (0.38 to 1.47) | 0.391 | 2.03 (0.91 to 4.55) | 0.085 |
|  | 36 mo | 4.9% (16/328) | 5.4% (18/335) | 5.4% (19/353) | 0.946 | 1.10 (0.58 to 2.11) | 0.766 | 1.00 (0.53 to 1.88) | 0.996 | 1.10 (0.57 to 2.12) | 0.773 |
|  | 36 mo, adjusted^a^ | - | - | - | 0.898 | 1.16 (0.59 to 2.29) | 0.659 | 1.03 (0.54 to 1.95) | 0.928 | 1.13 (0.58 to 2.20) | 0.717 |
|  | 36 mo, imputed^b^ | 4.8% | 5.4% | 5.3% | 0.926 | 1.11 (0.58 to 2.10) | 0.756 | 0.98 (0.53 to 1.81) | 0.949 | 1.13 (0.59 to 2.16) | 0.713 |
|  | 48 mo | 7.0% (23/331) | 8.5% (28/330) | 3.9% (13/333) | 0.059 | 0.56 (0.29 to 1.09) | 0.088 | 0.46 (0.24 to 0.87) | 0.018 | 1.22 (0.72 to 2.08) | 0.461 |
|  | 48 mo, adjusted^a^ | - | - | - | 0.178 | 0.66 (0.33 to 1.32) | 0.243 | 0.54 (0.28 to 1.04) | 0.063 | 1.24 (0.70 to 2.20) | 0.458 |
|  | 48 mo, imputed^b^ | 6.9% | 7.8% | 3.7% | 0.065 | 0.54 (0.28 to 1.04) | 0.066 | 0.48 (0.25 to 0.90) | 0.021 | 1.14 (0.68 to 1.93) | 0.620 |
|  | 60 mo | 9.1% (28/307) | 8.8% (27/308) | 5.9% (19/320) | 0.278 | 0.65 (0.37 to 1.14) | 0.134 | 0.68 (0.38 to 1.19) | 0.177 | 0.96 (0.58 to 1.59) | 0.878 |
|  | 60 mo, adjusted^a^ | - | - | - | 0.289 | 0.68 (0.37 to 1.25) | 0.217 | 0.63 (0.35 to 1.14) | 0.126 | 1.09 (0.64 to 1.84) | 0.758 |
|  | 60 mo, imputed^b^ | 8.9% | 8.9% | 6.2% | 0.330 | 0.70 (0.41 to 1.19) | 0.184 | 0.70 (0.41 to 1.19) | 0.183 | 1.00 (0.62 to 1.62) | 0.994 |

SP = sulfadoxine-pyrimethamine. AZI-SP = intervention group with monthly SP and two doses of azithromycin. NA = Not applicable, MUACZ is available only from three months of age onwards.

^a^ Adjusted for maternal malaria at enrollment, HIV status, height, body mass index, number of previous pregnancies, number of school years, and child sex.

^b^ Multiple imputation for missing data by chained equations and 50 imputations. SD for multiple imputed data calculated as an average SD from 50 imputations.

^c^ Estimates obtained from modified Poisson regression models.

^d^ Multiple imputation to replace weight measurements rounded to the full kilogram data by chained equations and 50 imputations. We considered all full kilogram weight measurements recorded at or after 36 months as censored within an interval of ± 0.5 kg of the full kilogram and used multiple imputation with interval censoring to replace the value. SD for multiple imputed data calculated as an average SD from 50 imputations.
