# Supplementary material for: The impact of maternal antenatal treatment with two doses of azithromycin and monthly sulphadoxine-pyrimethamine on child weight, mid-upper arm circumference and head circumference: A randomized controlled trial
Source: PLoS One. 2019 May 7;14(5):e0216536. doi: 10.1371/journal.pone.0216536 (PMC6504037; doi:10.1371/journal.pone.0216536)
Supplement: S7 Table — (DOCX) [file pone.0216536.s009.docx]

**Table S7. Mean (SD) weight-for-age Z-score (WAZ), weight-for-height Z-score (WHZ), mid-upper arm-circumference-for-age Z-score (MUACZ) and head circumference-for-age Z-score (HCZ) at last available time point by maternal malaria and HIV status at enrollment**

| **Outcome (number of negative / number of positive)** | **Mean (SD)** | | **Difference in means  (95% CI)** | **P-value** |
| --- | --- | --- | --- | --- |
|  | **Negative** | **Positive** |  |  |
| **Maternal HIV** |  |  |  |  |
| WAZ (965/144) | -1.30 (0.89) | -1.42 (1.07) | -0.12  (-0.30 to 0.06) | 0.197 |
| WHZ (965/144) | -0.37 (1.05) | -0.33 (1.13) | 0.04  (-0.16 to 0.23) | 0.707 |
| MUACZ (939/127) | -1.08 (0.84) | -1.14 (1.03) | -0.05  (-0.24 to 0.13) | 0.579 |
| HCZ (965/144) | -0.59 (1.05) | -0.62 (1.12) | -0.03  (-0.22 to 0.16) | 0.762 |
| **Maternal malaria** |  |  |  |  |
| WAZ (1122/105) | -1.30 (0.91) | -1.40 (0.96) | -0.10  (-0.29 to 0.09) | 0.292 |
| WHZ (1122/105) | -0.36 (1.07) | -0.40 (1.13) | -0.05  (-0.27 to 0.18) | 0.682 |
| MUACZ (1084/94) | -1.07 (0.87) | -1.03 (0.92) | 0.04  (-0.15 to -0.24) | 0.663 |
| HCZ (1122/105) | -0.57 (1.06) | -0.86 (1.00) | -0.29  (-0.50 to -0.09) | 0.004 |
