# Supplementary figures and images for: The impact of maternal antenatal treatment with two doses of azithromycin and monthly sulphadoxine-pyrimethamine on child weight, mid-upper arm circumference and head circumference: A randomized controlled trial
Source: PLoS One. 2019 May 7;14(5):e0216536. doi: 10.1371/journal.pone.0216536 (PMC6504037; doi:10.1371/journal.pone.0216536)

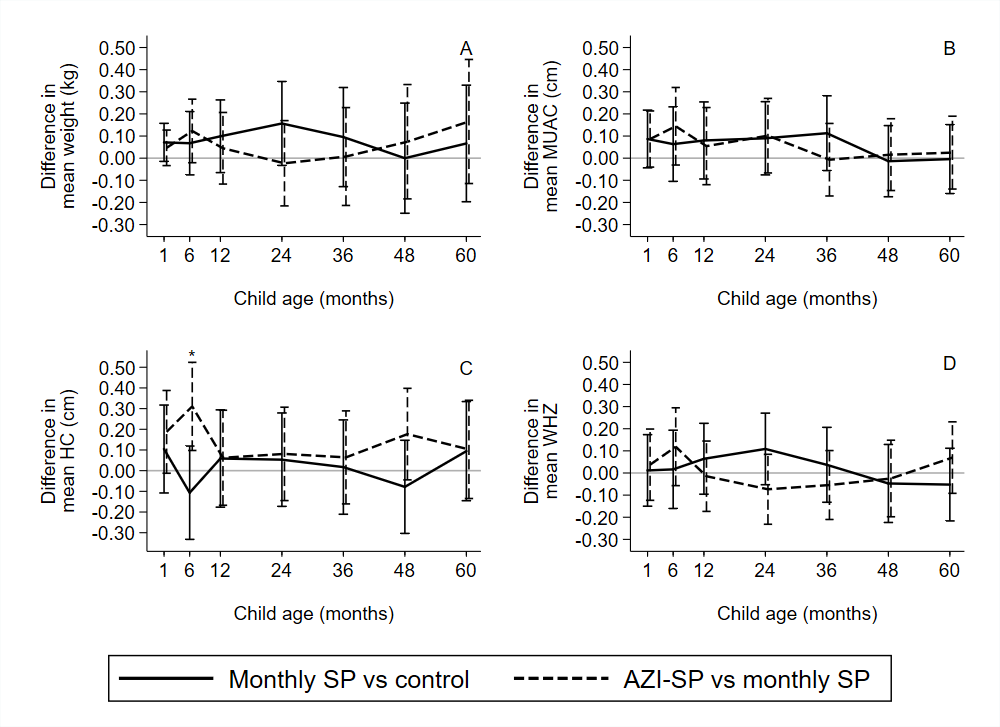

Supplement: S1 Fig — (A) Mean weight (kg). (B) Mean mid-upper arm circumference (MUAC). (C) Mean head circumference (HC). (D) Mean weight-for-height Z-score (WHZ). SP = sulfadoxine-pyrimethamine; AZI-SP = intervention group with monthly SP and two doses of azithromycin. * denotes global P<0.05. (TIF) [file pone.0216536.s010.tif]
